# Patients with anti-Jo1 antibodies display a characteristic IgG Fc-glycan profile which is further enhanced in anti-Jo1 autoantibodies

**DOI:** 10.1038/s41598-018-36395-z

**Published:** 2018-12-18

**Authors:** Cátia Fernandes-Cerqueira, Nuria Renard, Antonella Notarnicola, Edvard Wigren, Susanne Gräslund, Roman A. Zubarev, Ingrid E. Lundberg, Susanna L. Lundström

**Affiliations:** 1 Division of Rheumatology, Department of Medicine, Solna, Karolinska University Hospital, Karolinska Institutet, SE-171 76 Stockholm, Sweden; 2 Center for Molecular Medicine, Karolinska Institutet, Karolinska vägen 6 L8:04, SE- 171 76 Stockholm, Sweden; 3 Structural Genomics Consortium, Karolinska Institutet, Solnavägen 9, SE-171 77 Stockholm, Sweden; 4 Division of Physiological Chemistry I, Department of Medical Biochemistry and Biophysics, Karolinska Institutet, Solnavägen 9, SE- 171 77 Stockholm, Sweden

## Abstract

IgG Fc-glycans affect IgG function and are altered in autoimmune diseases and autoantibodies. Anti-histidyl tRNA synthetase autoantibodies (anti-Jo1) are frequent in patients with idiopathic inflammatory myopathies (IIM) and anti-synthetase syndrome (ASS) with associated interstitial lung disease (ILD). Thus, we hypothesized that the total-IgG Fc-glycans from Jo1^+^ versus Jo1^−^ patients and anti-Jo1-IgG would show characteristic differences, and that particular Fc-glycan features would be associated with specific clinical manifestations. By proteomics based mass spectrometry we observed a high abundance of agalactosylated IgG_1_ Fc-glycans in ASS/IIM patients (n = 44) compared to healthy age matched controls (n = 24). Using intra-individual normalization of the main agalactosylated glycan (FA2) of IgG_1_ vs FA2-IgG_2_, ASS/IIM and controls were distinguished with an area under the curve (AUC) of 79 ± 6%. For Jo1^+^ patients (n = 19) the AUCs went up to 88 ± 6%. Bisected and afucosylated Fc-glycans were significantly lower in Jo1^+^ compared to Jo1^−^ patients. Anti-Jo1-IgG enriched from eleven patients contained even significantly lower abundances of bisected, afucosylated and galactosylated forms compared to matched total-IgG. ASS and ILD diagnosis, as well as lysozyme and thrombospondin correlated with Jo1^+^ characteristic Fc-glycan features. These results suggest that the anti-Jo1^+^ patient Fc-glycan profile contains phenotype specific features which may underlie the pathogenic role of Jo1 autoantibodies.

## Introduction

Idiopathic inflammatory myopathies (IIM), myositis, are autoimmune conditions characterized by weakness and inflammation of skeletal muscle. Extra-muscular organs are commonly affected such as skin, joints and lung, the latter often as interstitial lung disease (ILD), being a major cause of morbidity and mortality^1,2^.

Autoantibodies are frequently found in patients with IIM, the most common being anti-Jo1 antibodies targeting histidyl transfer RNA synthetase (HisRS aka Jo1), which is an enzyme belonging to the family of aminoacyl-tRNA synthetases (aaRS). To date, HisRS and seven additional aaRS are recognized IIM-specific autoantigens. Autoantibodies targeting this family of enzymes (anti-Jo1, -PL7, -PL12, -EJ, -OJ, -KS, -Ha, -Zo) are, mainly monospecific, and associated with specific clinical manifestations: myositis, ILD, arthritis, Raynaud’s phenomenon, skin rash in the form of mechanic´s hands and fever^3^. Collectively, this clinical entity is termed anti-synthetase syndrome (ASS)^3^. Not all clinical settings coexist in the same patient and at the same time. However, up to 90% of patients with IIM and ILD present anti-Jo1 autoantibodies^4^.

**Figure 1:**
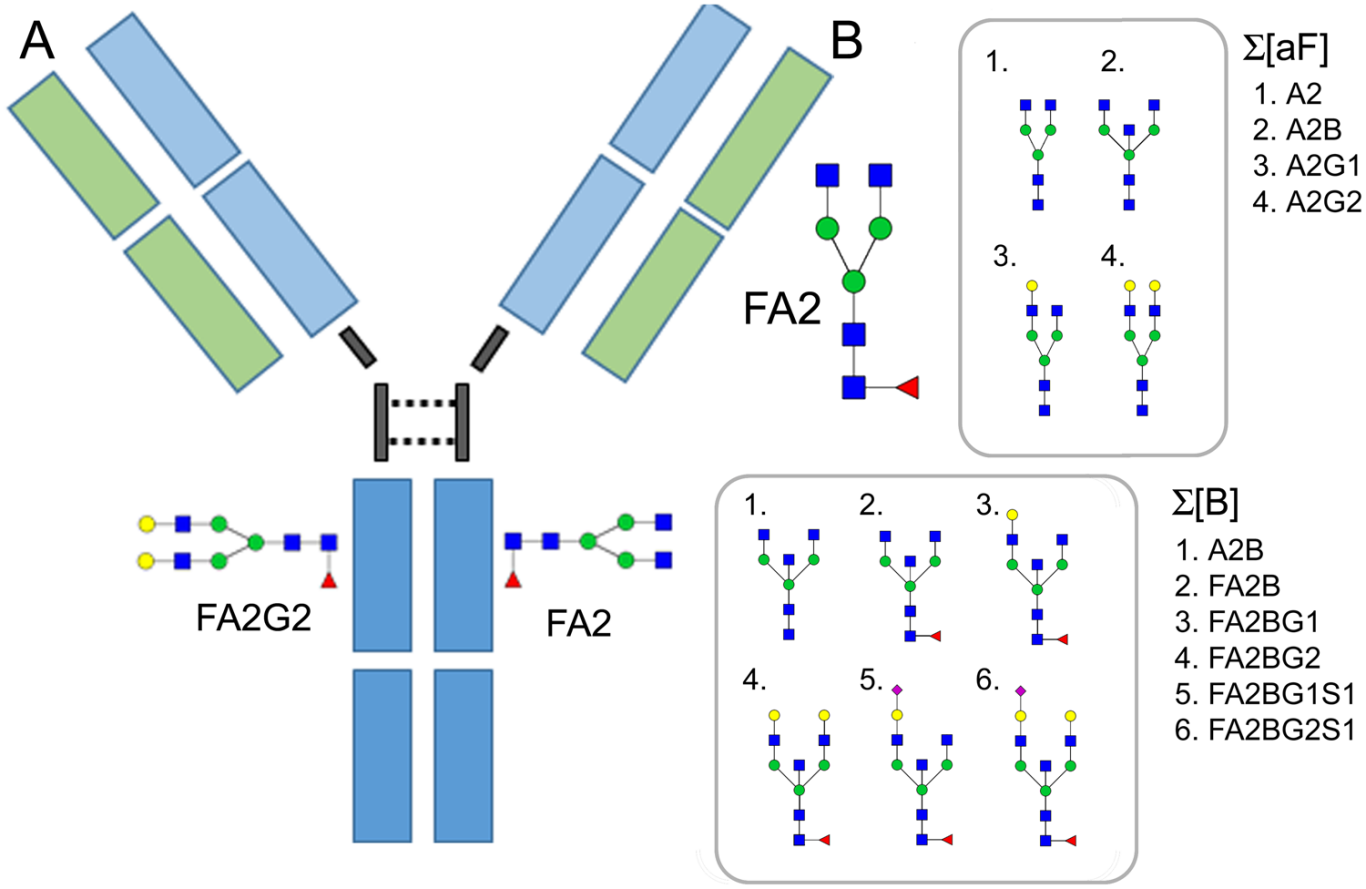
(**A**) Schematic figure of the IgG molecule and location of the Fc-glycans. Two common N-linked Fc-glycans, the digalactosylated (FA2G2) and the main agalactosylated form (FA2) are shown linked to the IgG-Fc region. (**B**) The factors (FA2, sum of afucosylated, Σ[aF] and sum of bisected forms Σ[B]) that were shown to affect the Fc-glycan profile of IIM or phenotype specific IIM most prominently. The glycan nomenclature is according to Royle *et al*.^44^. Blue squares (N-acetyl-glucosamine), red triangle (Fucose), green circles (Mannose), yellow circles (Galactose) and purple diamonds (Sialic Acid).

Accumulated evidence suggests that anti-Jo1 autoantibodies may be involved in the pathogenesis of ASS and/or IIM (ASS/IIM): serum levels of anti-Jo1 autoantibodies vary with disease activity^5,6^; and anti-Jo1 positive serum induces interferon (IFN) production from plasmacytoid dendritic cells^7^. However, direct evidence that anti-Jo1 autoantibodies cause or contribute to ASS/IIM pathogenesis is still lacking. One possible mechanism to explore is the effect of IgG Fc-glycans (Fig. 1). These sugars appended to the IgG Fc-region modulate inflammation by affecting the affinity of antibodies and interaction with Fcγ receptors (for example leading to antibody-dependent cellular cytotoxicity), engagement of complement activation, and induction of cytokine secretion^8^. Moreover, IgG Fc-glycosylation has been suggested as a possible disease biomarker, with low levels of galactosylation associated with a high pro-inflammatory status^9,10^. For instance, IgG_1_ Fc-galactosylation status predicts methotrexate (MTX) response in early rheumatoid arthritis (RA) patients^10^. Notably, Fc-glycans from anti-citrullinated protein autoantibodies (ACPA, known to induce pain and bone loss)^11,12^, are more agalactosylated and lack sialic acid residues, compared to non-ACPA IgG^13–15^. A significant decline in Fc-galactosylation and afucosylation of ACPAs has been reported prior to RA onset, which could suggest that Fc-glycosylation is a disease-driving mechanism^10,16^. In patients with IIM, more agalactosylated forms of IgG have likewise been found compared to healthy siblings and age/sex matched controls^17^. Together, these findings indicate the importance of antibody Fc-glycosylation for mediating immune regulating response and thus a potential role in pathogenicity of autoimmune disorders.

To better understand the potential role of anti-Jo1 autoantibodies in IIM pathogenesis we investigated the Fc-glycans of these autoantibodies as well as total IgG from anti-Jo1^+^ and anti-Jo1^−^ ASS/IIM patients. The Fc-glycan profiles were correlated with clinical manifestations and protein information (which was obtained by simultaneous proteomics screening of the IgG enriched samples).

## Results

### Number of patients, controls and sample types

In total, 24 healthy controls (HC) and 44 patients participated in the study. Of these patients, 15 were diagnosed with IIM but not ASS, 12 were diagnosed with ASS and IIM, one patient was diagnosed with ILD and IIM and 16 patients were diagnosed with ILD, ASS and IIM, (Table 1). 19 of the patients were Jo1 positive (Jo1^+^) and 25 Jo1 negative (Jo1^−^). For seven Jo1^−^ patients we had access to enriched sera IgG from two time points, (i.e. T1 and T2, Supplemental Table 1). For the Jo1^+^ patients we had access to enriched sera from two time points from six patients, three time points from two patients, as well as one patient with seven, one patient with eight and one patient with ten sampling time points (i.e. T1–T10), Supplemental Table 1. Note that the intra-individual glycan profiles of these patients did not change significantly over time (for more details see Supplemental Fig. 1). For this reason, we are focusing on the first sampling time point (T1) for which we have data from all patients. For extra validation both univariate and multivariate data were evaluated by including the remaining data from the other time points. From nine Jo1^+^ patients, anti-Jo1 auto-immune IgG was further enriched from the total serum IgG from respective patient using either pools from several sampling time points per patient (n = 8), or from one sampling time point (n = 1). Furthermore, we had access to anti-Jo1 specific IgG from two additional patients both sampled at one occasion. For statistical comparisons, the patients were sub-grouped according to ASS and/or IIM (ASS/IIM), ILD and/or ASS (ILD/ASS), Jo1^−^ and Jo1^+^, respectively (Table 1). The auto-immune enriched anti-Jo1 IgG was compared to the intra-individual differences relative to the IgG prior enrichment (PE) and the IgG deprived from anti-Jo1 IgG (Flow Through, FT), Table 1.

**Table 1:** Overview of the sample types and patient sub-groupings. Note that from eleven Jo1^+^ patients, anti-Jo1 auto-immune IgG was further enriched. Of these 9 were from patients already included in the study by accessible IgG enriched from serum. Additionally, we had access to anti-Jo1 specific IgG from two more patients, (i.e. 9 + 2)).

Type	Label	Control	IIM	ASS and IIM	ILD and IIM	ASS, ILD and IIM	Total
Total IgG	HC	24	—	—	—	—	24
(Enriched IgG from serum)	ASS/IIM	—	15	12	1	14	42
	ILD/ASS	—	—	12	1	14	27
	Jo1^−^		15	9	1	—	25
	Jo1^+^		—	3	—	14	17
anti-Jo1 IgG (Enriched from Total	anti-Jo1	—	—	—	—	9 + 2	11
IgG), and intra-individually	PE (Prior Enrichment IgG)	—	—	—	—	9 + 2	11
matched PE-IgG and FT-IgG	FT (Flow Through IgG)	—	—	—	—	9 + 2	11

HC; Healthy Control, IIM; idiopathic inflammatory myopathies, ASS; anti-synthetase syndrome, ILD; interstitial lung disease, Jo1^−^; Jo1 negative, Jo1^+^; Jo1 positive.

### Patient demographics

Clinical characteristics of ASS/IIM patients are summarized in Table 2 and Supplementary Table 2. The frequency of ILD in Jo1^+^ patients (84%) was significantly higher than in Jo1^−^ (36%, p = 0.002). A larger (non-significant, p = 0.07) percentage of Jo1^+^ patients (58%) were diagnosed with arthritis compared to Jo1^−^ patients (28%).

**Table 2:** Demographic data at first available serum sample.

	Total IIM (n = 44)	Anti-Jo1^+^ (n = 19)	Anti-Jo1^−^ (n = 25)
Age, mean years (SD)	58.6 (11.7)	54.5 (11.2)	61.8 (11.1)
Women, n (%)	24 of 44 (54.5)	9 of 19 (47.4)	15 of 25 (60.0)
PM/DM/IBM, %	65/30/5	79/21/0	56/36/8
Disease duration in months, median (25–75^th^ percentiles)	24 (0–60)	36 (12–60)	12 (0–54)
Anti-synthetase syndrome, n (%)	28 (63.6)	19 of 19 (100)	9 of 25 (36.0)^a^
**Extra-muscular manifestations**, **n (%)**			
Interstitial Lung Disease, n (%)	25 (58.1)	16 of 19 (84.2)	9 of 25 (36.0)^**b**^
Arthritis	18 of 44 (40.9)	11 of 19 (57.9)	7 of 25 (28.0)
Dysphagia	9 of 44 (20.5)	3 of 19 (15.8)	6 of 25 (24.0)
Skin rash	14 of 44 (31.8)	5 of 19 (26.3)	9 of 25 (36.0)
Smoking status, n ever (%)	24 of 44 (54.5)	10 of 19 (52.6)	14 of 25 (56.0)
**Laboratory tests**			
CK, median µcat/L (25–75^th^ percentiles)	3.4 (1.3–12.7)	1.3 (1–7.9)	4.4 (1.7–15.7)^**c**^
**Antibodies**			
Positive anti-PL7, n (%)	2 (5.1)	0	2 (8.3)
Positive anti-PL12, n (%)	2 (5.1)	0	2 (8.3)
Positive anti-EJ, n (%)	1 (2.5)	0	1 (4.2)
Positive anti-OJ, n (%)	3 (7.7)	0	3 (12.5)
Positive anti-Mi-2, n (%)	3 (7.9)	1 (7.1)	2 (8.3)
Positive anti-SRP, n (%)	2 (5.1)	0	2 (8.3)
Positive anti-MDA5, n (%)	3 (7.9)	0	3 (12.5)
Positive anti-TIF1g, n (%)	3 (7.9)	0	3 (12.5)
Positive anti-SSA, n (%)	16 (36.4)	10 (52.6)	6 (24.0)
Positive anti-SSB, n (%)	0	0	0
Positive anti-U1 RNP, n (%)	5 (11.4)	2 (10.5)	3 (12.0)
Positive anti-Ku, n (%)	1 (2.5)	0	1 (4)
Positive anti-PmScl, n (%)	2 (4.9)	1 (6.3)	1 (4)
Physician VAS, median (25–75^th^ percentiles)	37 (10–50)	37 (0–60)	35 (12–50)
Patient VAS, median (25–75^th^ percentiles)	28 (8–52)	21 (6–47)	32 (8–55)
MDAAT, median (25–75^th^ percentiles)	0.06 (0–0.15)	0.07 (0–0.16)	0.06 (0–0.15)
HAQ (1–3), median (25–75^th^ percentiles)	0.75 (0–1.41)	0.63 (0.13–1.25)	1 (0–1.5)
MMT-8 (0–80), median (25–75^th^ percentiles)	78 (68–80)	80 (77–80)	75 (64–80)^**d**^
**Immunosuppressive (IS) treatment**, n (%)*			
No treatment	10 (23)	4 (21)	6 (24)
1 treatment	12 (27)	3 (16)	9 (36)
2 or 3 concomitant treatments	22 (50)	12 (63)	10 (40)
**Healthy controls (n** **=** **24)**			
Age, mean years (SD)	59.3 (13.0)		
Women, n (%)	12 of 24 (50)		

IIM, idiopathic inflammatory myopathies; VAS physician, physician’s global disease activity assessment; VAS patient, patient’s global disease activity assessment; MDDAT, Myositis Disease Activity Assessment Tool for extramuscular global assessment; HAQ, Health Assessment Questionnaire; MMT-8, Manual Muscle Testing; *1 treatment designates one of the following treatments alone: methotrexate, glucocorticoids, intravenous Ig, abatacept or azathioprine (AZA); 2 or 3 concomitant treatments designate all the possible following combinations: glucocorticoids + AZA, glucocorticoids + cyclophosphamide, glucocorticoids + methotrexate, glucocorticoids + mycophenolate mofetil, glucocorticoids + rituximab, glucocorticoids + cyclophosphamide+ rituximab, glucocorticoids + methotrexate +rituximab, or glucocorticoids + mycophenolate mofetil+rituximab. Missing information on VAS physician: 5 anti-Jo1^−^ and 4 anti-Jo1^+^ patients; Missing information on VAS patient: 6 anti-Jo1^−^ and 4 anti-Jo1^+^; Missing information on MDAAT: 10 anti-Jo1^−^ and 6 anti-Jo1^+^; Missing information on HAQ: 6 anti-Jo1^−^ and 4 anti-Jo1^+^; Missing information on MMT-8: 4 anti-Jo1^−^ and 3 anti-Jo1^+^. ^a^p < 0.001; ^b^p = 0.002 vs anti-Jo1^+^ (Fisher’s test); ^c^p = 0.018 vs anti-Jo1^+^ and ^d^p = 0.015 vs anti-Jo1^+^ (Mann-Whitney test).

### Total ASS/IIM IgG and anti-Jo1-IgG serum concentration and reactivity

Efficient purification of total IgG and anti-Jo1-IgG was confirmed by well-defined protein gel bands localized in the antibody heavy and light chain regions of total IgG and anti-Jo1-IgG (Supplementary Fig. 2C). An ELISA and a dotblot were performed in order to confirm the efficiency of anti-Jo1 IgG enrichment from the total IgG loaded into the Jo1 affinity column (Supplementary Fig. 2C; Supplementary methods). Strong anti-Jo1 reactivity was registered by anti-Jo1-IgG, and no major reactivity against Jo1 was detected in the remaining control flow through IgG (FT; Supplementary Fig. 2B and D).

Total IgG concentration (median < 8.6 mg/mL) in serum and IgG percentage among total serum proteins (median < 16.6 mg/mL) at first available sample was similar in patients and controls (Supplementary Table 3).

The median concentration of anti-Jo1-IgG in ASS/IIM was 0.06 mg/mL, reaching a maximum of 0.13 mg/mL. The proportion of anti-Jo1-IgG among total IgG in ASS/IIM serum was 1.58%, with 4 out of 11 patients showing a percentage greater than 2% (Supplementary Table 3). No significant changes of IgG titres were observed in longitudinally collected sera (Supplementary Table 4).

### ASS/IIM patients display a significantly less galactosylated IgG_1_ Fc-glycan profile compared to healthy controls

**Figure 2:**
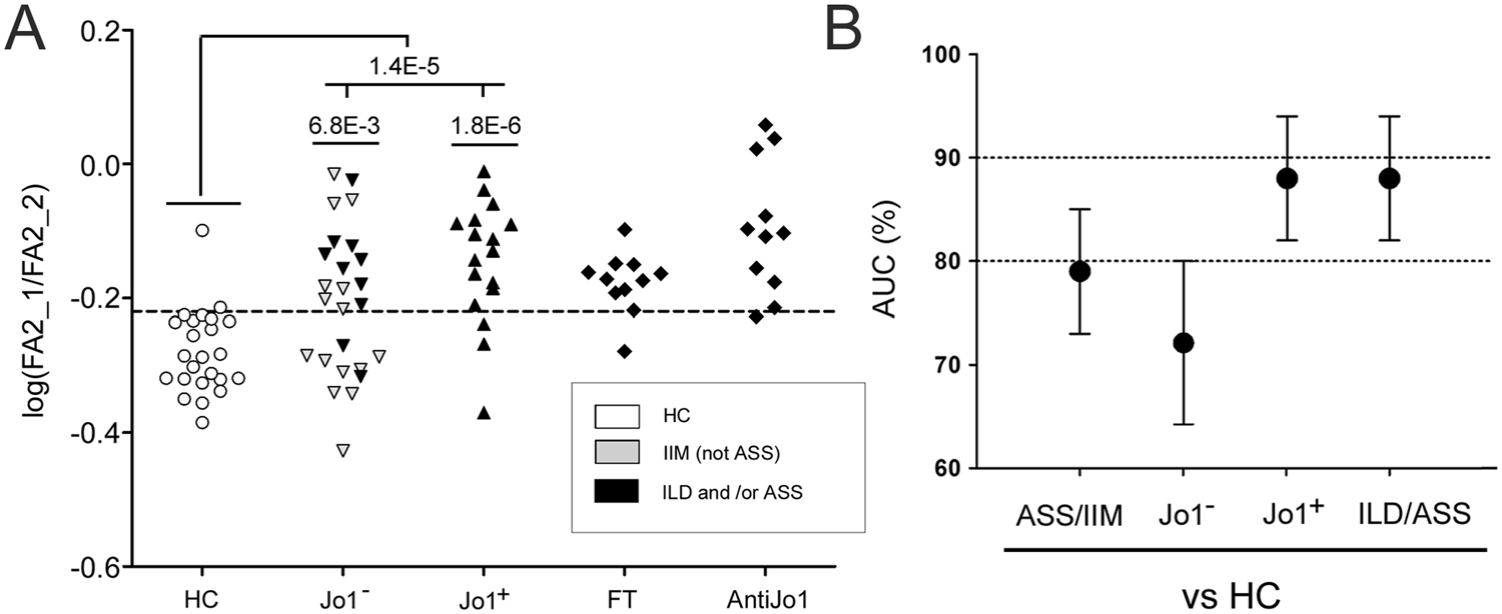
(**A**) The variation in log(FA2_1/FA2_2) in healthy controls (HC), and ASS/IIM (Jo1^−^ and Jo1^+^) patients as well as in anti-Jo1 enriched IgG and in matched flow through (FT) from Jo1^+^ patients. At the cut off of -0.22, ASS/IIM could be separated from the controls with a 69% sensitivity and 92% specificity. For Jo1^+^ patients and for ILD and/or ASS (ILD/ASS) diagnosed patients (coloured in black) the sensitivity further increased to 82%. (**B**) AUC values of ROC-curve analyses obtained comparing the controls to the ASS/IIM according to different subgroups. Error bars represent 95% confidence intervals. P-values < 0.005 remain significant following FDR correction.

Eighteen Fc-glycans from IgG_1_, sixteen from IgG_2/(3)_ and twelve from IgG_3/4_ were quantified. The glycoform abundances were normalized to total content (100%) of Fc-glycosylated IgG_1_, total content (100%) of Fc-glycosylated IgG_2/(3)_ and total content (100%) of Fc-glycosylated IgG_4/(3)_ peptides, respectively. In Supplementary Table 5, the individual average ± standard deviations for each glycopeptide, as well as p-values (comparing the subgroups listed in Table 1) are given. Comparing ASS/IIM with controls, specifically the ASS/IIM Fc-glycan profile of IgG_1_ was altered with half of the glycans being significantly different (p < 0.05). Of these significant glycans seven (out of nine) indicated a decrease in galactosylation. In contrast, the galactosylation status of IgG_2/(3)_ and of IgG_3/4_ remained relatively stable. For example, compared to the main agalactosylated form FA2 (Fig. 1) from IgG_1_ which was significantly increasing (p = 4.5E-4), FA2 of IgG_2/(3)_ and FA2 of IgG_3/4_ were not significantly different between the controls and the ASS/IIM patients (p = 1.9E-1 and p = 6.4E-2, respectively), Supplementary Table 5. Since the galactosylation status is known to change to less galactosylated forms, not only according to inflammatory/disease status, but also according to factors such as age, sex and heritability^18^, we tested if the unchanged FA2 of IgG_2/(3)_ (FA2_2) could be used as an intra-individual control of the change in FA2 of IgG_1_ (FA2_1). Thus, we used log(FA2_1/FA2_2), and tested how well this factor could distinguish patients with ASS/IIM compared to controls. In terms of significance, log(FA2_1/FA2_2) was significantly different (p = 1.4E-5, Table 3 and Fig. 2A). Furthermore, ROC curve analysis of log(FA2_1/FA2_2) generated an area under the curve (AUC) of 79 ± 6% when comparing ASS/IIM to controls. Noteworthy, when selecting and comparing the subgroups of Jo1^+^ patients to controls and Jo1^−^ patients to controls separately, the AUC went up to 88 ± 6% for Jo1^+^ patients and down to 72 ± 8% for Jo1^−^ patients (Fig. 2B). An AUC of 88 ± 6% was also reached for the subgroup of patients that had an ILD and/or ASS (ILD/ASS) diagnosis (independent on Jo1 reactivity, n = 28, Fig. 2A,B). At a cut off of −0.22, 69% sensitivity and 92% specificity was obtained for all patients (n = 42) or separately 82/92% for all Jo1^+^ patients as well as for all ILD/ASS diagnosed patients, and 60/92% for all Jo1^−^ patients.

### Jo1^+^ patients display an Fc-glycan profile with less bisected and afucosylated glycans compared to Jo1^−^ patients

While the main differences between controls and ASS/IIM patients were observed in IgG_1_ Fc-galactosylation status, particularly for the most abundant forms FA2 (increasing) as well as FA2G1 and FA2G2 (decreasing), the most prominent differences in the Fc-glycan profiles between Jo1^+^ and Jo1^−^ patients were observed in significantly lower abundances of bisected (6 out of 16) and afucosylated (2 out of 7) glycopeptides, Supplementary Table 5. These differences were not IgG_1_ specific with particularly the bisected and galactosylated forms of IgG_1_ and IgG_2_ (FA2BG1, p = 4.9E-3 and 1.5E-3 as well as FA2BG2, p = 1.9E-3 and p = 1.3E-2, Supplemental Table 5), being significantly different. To validate the decline in afucosylation and bisection in Jo1^+^ patients and to overall test the influence of different Fc-glycan features on the patient profiles, the isotype specific glycans were grouped and statistically compared (Table 3). Hence, the different glycan features were compared according to the sum of galactosylated Σ[G], sum of agalactosylated Σ[aG], sum of afucosylated Σ[aF], sum of bisected Σ[B], and sum of sialylated Σ[S] forms. The subgrouping of Σ[aF] and of Σ[B] are shown in Fig. 1B. Using this approach, it was confirmed that the Jo1^+^ patients have lower abundance of bisected (p = 2.9E-3) and afucosylated (p = 4.8E-2) forms (Table 3, Supplemental Fig. 3). The trend of these differences was observed for all IgG isotypes and was most prominent for the sum of bisected IgG_2/(3)_ glycans (p = 9.7E-4) and afucosylated IgG_1_ glycans (p = 3.3E-2) (Table 3, Supplementary Fig. 3).

**Table 3:** Fc-glycan distributions (%, average ±  SD) and p-values for group comparisons according to glycan type (i.e. galactosylated: containing galactose [G], agalactosylated: lacking galactose [aG]), afucosylated: lacking fucose [aF], bisected: containing glycan bisection [B], and sialylated: containing sialic acid [S]) for the different subgroups. Additionally the galactosylation status was measured via log(FA2_1/FA2_2), i.e. the intra individual differences of FA2 on IgG_1_ and IgG_2/(3)_. P-values < 0.005 (5.0E-3, bold numbers) remain significant following FDR correction.

Isotype	Glycan factor	T1	IIM	Jo1^−^	Jo1^+^	Jo1^+^ enrichment		Anti-Jo1	Healthy vs						IIM			
						PE	FT		IIM	All T	Jo1^−^		Jo1^+^		Jo1^−^/Jo1^+^		Anti-Jo1 vs	
		HC							T1		T1	All T	T1	All T	T1	All T	FT	PE
IgG_1_/IgG_2/(3)_	log(FA2_1/FA2_2)	−0.28 ± 0.06	−0.18 ± 0.11	−0.20 ±0.11	−0.15±0.09	−0.16±0.08	−0.18 ± 0.05	−0.09 ± 0.10	**1**.**4E**−**05**	**6**.**1E**−**06**	6.8E−03	1.2E−02	**1**.**8E**−**06**	**7**.**3E**−**07**	6.3E−02	6.3E−02	1.3E−02	9.5E−02
IgG1	Σ[aG_1], n = 5	27 ± 9	37 ± 13	35 ± 13	41 ± 12	41 ± 9	39 ± 4	47 ± 10	**1**.**3E**−**03**	**2**.**3E**−**04**	2.0E−02	2.7E−02	**2**.**2E**−**04**	**6**.**4E**−**06**	1.9E−01	1.2E−01	3.6E−02	2.2E−01
	Σ[G_1], n = 12	72 ± 9	62 ± 13	64 ± 14	59 ± 12	58 ± 9	60 ± 4	53 ± 10	**3**.**8E**−**04**	**2**.**5E**−**04**	1.9E−02	2.8E−02	**1**.**7E**−**04**	**6**.**8E**−**06**	1.9E−01	1.3E−01	3.7E−02	2.2E−01
	Σ[S_1], n=5	16 ± 7	13 ± 6	13 ± 6	12 ± 6	10 ± 3	10 ± 4	9 ± 5	3.9E−02	3.1E−02	1.2E−01	5.7E−02	5.6E−02	1.2E−02	4.8E−01	7.4E−01	7.9E−01	6.6E−01
	Σ[B_1], n = 6	23 ± 6	24 ± 6	25 ± 6	22 ± 6	25 ± 5	24 ± 4	21 ± 6	7.2E−01	5.2E−01	3.4E−01	3.4E−01	5.7E−01	7.8E−01	1.5E−01	2.8E−01	1.0E−02	**2**.**2E**−**03**
	Σ[aF_1], n = 4	4 ± 2	4 ± 2	5 ± 2	3 ± 2	4 ± 1	4 ± 2	2 ± 2	3.6E−01	2.6E−01	6.7E−02	**5**.**2E**−**03**	4.7E−01	5.1E−01	**3**.**3E**−**02**	**3**.**9E**−**04**	**1**.**7E**−**03**	6.7E−02
IgG2/(3)	Σ[aG_2], n = 5	44 ± 11	48 ± 11	48 ± 10	50 ± 12	51 ± 12	52 ± 8	53 ± 12	1.5E−01	3.5E−02	2.8E−01	2.0E−01	1.6E−01	2.1E−02	5.9E−01	2.7E−01	6.2E−01	3.5E−01
	Σ[G_2], n = 10	56 ± 11	51 ± 11	52 ± 10	50 ± 12	49 ± 12	47 ± 8	46 ± 12	1.4E−01	3.5E−02	2.7E−01	1.9E−01	1.6E−01	2.1E−02	6.0E−01	2.9E−01	6.2E−01	3.5E−01
	Σ[S_2], n=5	9 ± 5	8 ± 4	8 ± 4	9 ± 6	8 ± 4	4 ± 3	9 ± 4	4.2E−01	1.8E−01	3.3E−01	2.1E−01	7.0E−01	2.4E−01	7.0E−01	1.0E + 00	6.0E−03	2.6E−01
	Σ[B_2], n = 6	16 ± 3	16 ± 4	18 ± 3	14 ± 3	15 ± 3	16 ± 3	13 ± 4	9.0E−01	9.7E−01	1.7E−01	1.3E−01	3.4E−02	2.0E−01	**9**.**7E**−**04**	**2**.**0E**−**03**	**1**.**7E**−**03**	3.7E−02
	Σ[aF_2], n = 2	1 ± 1	1 ± 1	2 ± 1	1 ± 1	1 ± 1	1 ± 1	1 ± 1	5.1E−02	1.5E−02	4.0E−02	**1**.**5E**−**03**	4.4E−01	3.1E−01	3.1E−01	1.1E−02	8.4E−01	9.5E−01
IgG(3)/4	Σ[aG_34], n = 4	45 ± 12	49 ± 12	50 ± 12	47 ± 12	45 ± 9	45 ± 9	48 ± 11	1.7E−01	1.1E−01	1.2E−01	2.3E−02	5.1E−01	5.3E−01	4.5E−01	9.6E−03	1.1E−01	5.1E−02
	Σ[G_34], n = 7	55 ± 12	51 ± 12	50 ± 12	52 ± 12	55 ± 9	55 ± 9	51 ± 10	1.7E−01	1.2E−01	1.3E−01	2.5E−02	5.0E−01	4.9E−01	4.8E−01	1.0E−02	1.0E−01	3.9E−02
	Σ[S_34], n = 2	13 ± 6	10 ± 5	10 ± 4	11 ± 6	10 ± 4	9 ± 3	11 ± 4	5.3E−02	7.7E−03	3.7E−02	1.2E−02	2.9E−01	4.6E−02	5.0E−01	1.9E−01	1.8E−01	7.5E−01
	Σ[B_34], n = 4	25 ± 7	24 ± 6	26 ± 5	21 ± 5	22 ± 6	21 ± 6	27 ± 8	6.6E−01	4.6E−01	4.5E−01	2.2E−01	7.1E−02	2.4E−02	**4**.**9E**−**03**	**1**.**5E**−**05**	4.7E−02	9.5E−03
	Σ[aF_34], n = 1	0.4 ± 0.4	0.4 ± 0.4	0.4 ± 0.4	0.4 ± 0.5	0.3 ± 0.4	0.3 ± 0.3	0.2 ± 0.2	8.7E−01	6.4E−01	8.0E−01	9.4E−01	9.9E−01	4.1E−01	8.2E−01	2.9E−01	6.6E−01	7.2E−01
IgG (combined)	Σ[aG_total], n = 14	117 ± 30	135 ± 32	133 ± 32	138 ± 33	137 ± 23	137 ± 17	148 ± 26	2.6E−02	6.0E−03	7.0E−02	3.2E−02	4.1E−02	6.5E−03	6.8E−01	9.5E−01	6.3E−02	1.7E−01
	Σ[G_total], n = 29	183 ± 30	164 ± 32	166 ± 33	161 ± 33	162 ± 23	163 ± 17	151 ± 26	2.5E−02	6.4E−03	6.9E−02	3.3E−02	3.8E−02	6.8E−03	6.6E−01	9.7E−01	5.9E−02	1.6E−01
	Σ[S_total], n = 12	39 ± 15	31 ± 13	31 ± 11	32 ± 16	28 ± 8	23 ± 9	29 ± 9	4.5E−02	6.8E−03	5.5E−02	1.1E−02	1.6E−01	2.3E−02	9.5E−01	7.3E−01	9.3E−02	8.5E−01
	Σ[B_total], n = 16	64 ± 13	64 ± 12	68 ± 11	57 ± 12	62 ± 13	61 ± 10	61 ± 15	9.4E−01	9.4E−01	2.2E−01	1.4E−01	9.5E−02	1.8E−01	**2**.**9E**−**03**	**2**.**9E**−**04**	9.5E−01	7.6E−01
	Σ[aF_total], n = 7	5 ± 2	6 ± 3	7 ± 3	5 ± 3	5 ± 2	6 ± 3	4 ± 2	2.2E−01	2.2E−01	3.6E−02	**1**.**4E**−**03**	7.8E−01	7.5E−01	4.8E−02	**1**.**4E**−**04**	2.1E−02	1.5E−01

Abbreviations: HC: Healthy control, IIM: idiopathic inflammatory myopathies, Jo1^−^: Jo1 negative, Jo1^+^: Jo1 positive, PE: IgG Prior Enrichment of anti-Jo1-IgG, FT: Flow Through, anti-Jo1: anti-Jo1 specific IgG, T1: Time point 1, T total: data from all timepoints (T1–T10). Glycan abbreviations are described in Fig. 1. Σ[aG_1]: Σ[IgG1: A2, A2B, FA1, FA2 and FA2B], Σ[G_1]: [IgG1: A2G1, A2G2, FA1G1, FA1G1S1, FA2G1, FA2G2, FA2G1S1, FA2G2S1, FA2BG1, FA2BG2, FA2BG1S1 and FA2BG2S1], Σ[S_1]: [IgG1:FA1G1S1, FA2G1S1, FA2G2S1, FA2BG1S1 and FA2BG2S1], Σ[B_1]: [IgG1: A2B, FA2B, FA2BG1, FA2BG2, FA2BG1S1 and FA2BG2S1], Σ[aF_1]: [IgG1: A2, A2B, A2G1 and A2G2], Σ[aG_2]: [IgG2: A2, A2B, FA1, FA2 and FA2B], Σ[G_2]: [IgG2: FA1G1, FA1G1S1, FA2G1, FA2G2, FA2G1S1, FA2G2S1, FA2BG1, FA2BG2, FA2BG1S1 and FA2BG2S1], Σ[S_2]: [IgG2: FA1G1S1, FA2G1S1, FA2G2S1, FA2BG1S1 and FA2BG2S1], Σ[B_2]: [IgG2: A2B, FA2B, FA2BG1, FA2BG2, FA2BG1S1 and FA2BG2S1], Σ[aF_2]: [IgG2: A2 and A2B], Σ[aG_34]: [IgG34: A2B, FA1, FA2 and FA2B], Σ[G_34]: [IgG34: FA1G1, FA2G1, FA2G2, FA2G1S1, FA2G2S1, FA2BG1 and FA2BG2], Σ[S_34]: [IgG34: FA2G1S1 and FA2G2S1], Σ[B_34]: [IgG34: A2B, FA2B, FA2BG1 and FA2BG2], Σ[aF_34]: [IgG34: A2B], Σ[aG_total]: sum all agalactosylated, Σ[G_total]:sum all galactosylated, Σ[S_total]: sum all sialylated, Σ[B_total]:sum all bistected, Σ[aF_total]:sum all afucosylated. For individual glycan values see Supplementary Table 5.

### The Fc-glycan profile of anti-Jo1 specific IgG is an enhanced version of the Fc-glycan profile observed in total IgG from Jo1^+^ patients

**Figure 3:**
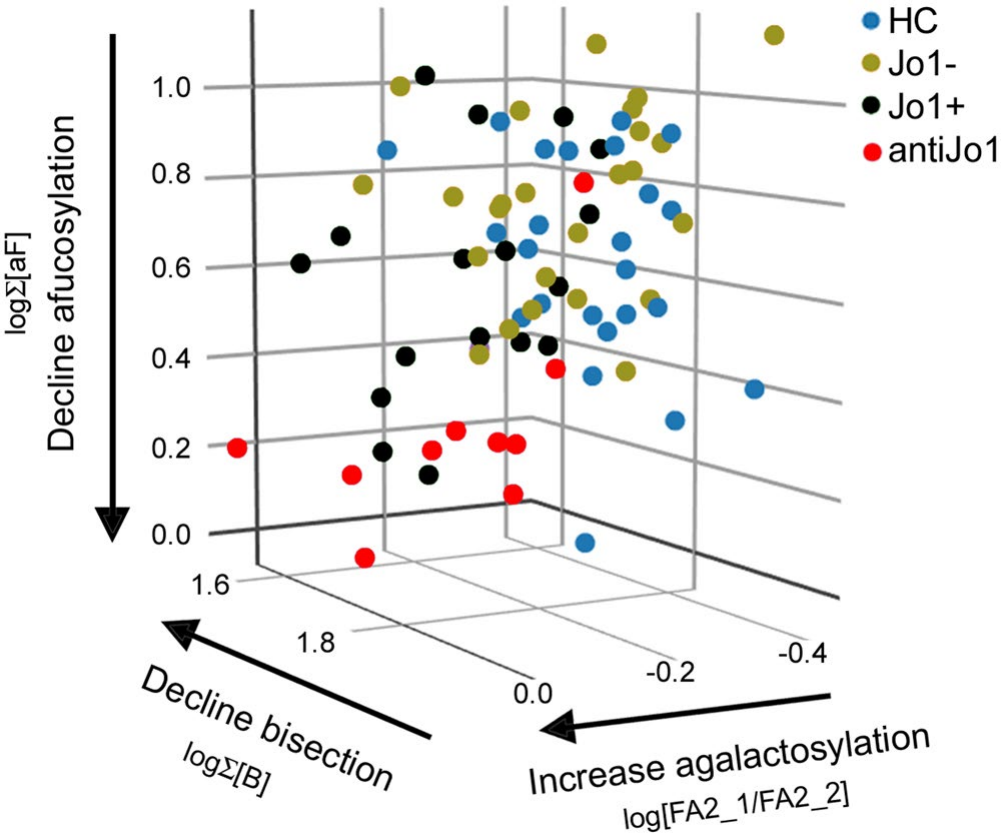
3D-plot of the three factors (agalactosylation, bisection, and afucosylation) that best distinguished the anti-Jo1 specific IgG (red circles) and Jo1^+^ patients total IgG (black circles) from both total IgG from controls (blue circles), and from Jo1^−^ patients (green circles). Thus, the anti-Jo1 specific IgG and the Jo1^+^ patients total IgG has 1) an increase in IgG_1_ agalactosylation (described by log(FA2_1/FA2_2) and 2) a decline in bisected forms (described by log Σ[B]) and 3) a decline in afucosylated forms (described by logΣ [aF]).

In a next step the Fc-glycans from the disease specific anti-Jo1 autoimmune reactive IgG (enriched from the IgG from the Jo1 positive patients) were interrogated and compared intra-individually to the IgG prior to anti-Jo1 enrichment, (“PE” in Table 3) and to the remaining non-anti-Jo1 specific IgG following enrichment (termed flow through, FT in Table 3). Strikingly, the anti-Jo1 specific Fc-glycans had a similar (but enhanced profile) of what was characteristic for the total-IgG profile of Jo1^+^ patients. Thus, the Fc-glycan profiles of anti-Jo1 specific IgG were generally the least bisected, least afucosylated and most agalactosylated compared to the total IgG from Jo1^+^ patients and subsequently compared to the IgG from Jo1^−^ patients and the controls (Table 3, Supplemental Figs 3 and 4). In Fig. 3 this trend is visualised by plotting the three factors (i.e. agalactosylation: described by log(FA2_1/FA2_2), afucosylation: described by Σ[aF] and bisection: described by Σ[B] from the total-IgG from controls, Jo1^−^ and Jo1^+^ patients, and from the anti-Jo1 specific IgG. Clearly, anti-Jo1 enriched IgG clusters together (red circles) in one corner of the 3D plot (due to the highest abundance of agalactosylated and lowest abundance of afucosylated and bisected Fc-glycans). The Jo1^+^ patients total-IgG (black circles) are closest in space to the specific anti-Jo1-IgG (red circles). Inversely, the Jo1^−^ patients total-IgG (yellow circles) and control total-IgG (blue circles) are furthest away from the anti-Jo1-IgG cluster.

### Multivariate correlation analyses of the Fc-glycan patient profiles

Multivariate statistical modelling (MVA) was done to interrogate how well features within the Fc-glycan profile would correlate with clinical and diagnostic patient information and protein data obtained from the IgG enriched samples. Two MVA models were created and assessed to distinguish (1) ASS/IIM (compared to controls) and (2) Jo1^+^ patients (compared to Jo1^−^ patients). For details of the ASS/IIM vs control analysis, see Supplementary material.

### MVA correlation analysis distinguishing Jo1^+^ vs Jo1^−^ patient features

**Figure 4:**
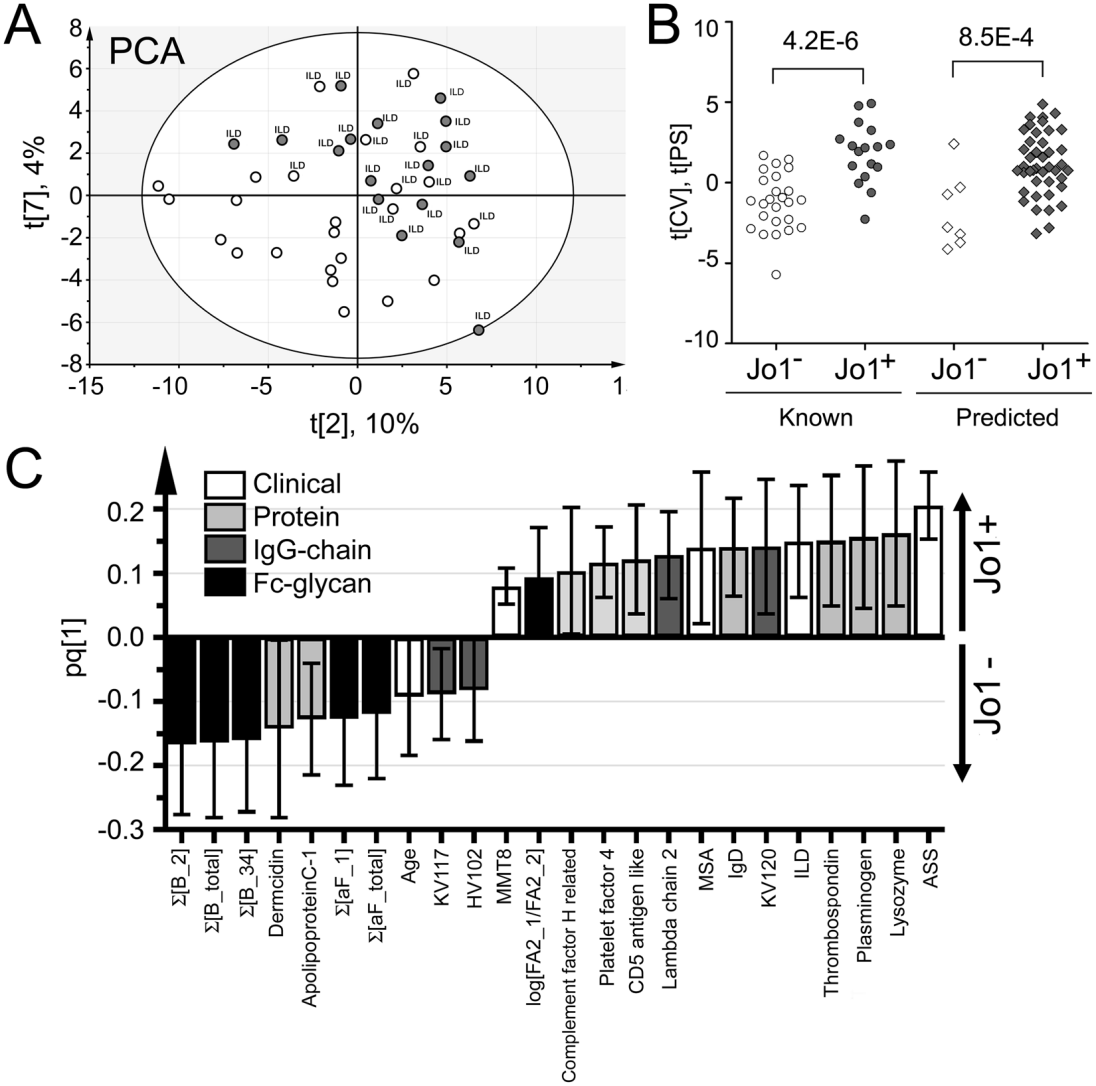
Multivariate correlation analyses according to Jo1^+^ patient selection including the proteomics data analysis of the samples. (**A**) PCA scores plot. The Jo1^+^ patients and/or patients with ILD diagnosis cluster together along component 2 (x-axis) and component 7 (y-axis). This indicates that there are factors that are characteristic for this patient group within the model. (**B**) OPLS-DA dot plot of the cross validated (tCV) scores (circles) and of predicted scores (diamonds) obtained from the model set to identify Jo1^+^ correlating features in the data set (all factors are listed in Supplementary Table 6). Both the Jo1^+^ and Jo1^−^ patient profiles of time point 1 that were treated as known (i.e. from which the model was based of) and unknown (the validation cohort of patients profiles analysed at other time points), are significantly different. (**C**) The factors that correlated positively or negatively with Jo1^+^ patients with 95% confidence in the OPLS-DA model.

In Fig. 4A, a PCA model (R^2^ = 0.53, 8 components) of the ASS/IIM patient data is presented. Noteworthy, a majority of the Jo1^+^ patients (dark circles) cluster in one part of the plot. This clustering indicates that there are features within the model that are similar for these patients and different from the Jo1^−^ patient subgroup (white circles). However, there are also some overlap between the two subgroups. Noteworthy, these “outliers” are Jo1^−^ patients with ILD symptoms. To distinguish which factors most prominently separated the Jo1^+^ and Jo1^−^ patient, an OPLS-DA model of the data was created (R^2^ = 0.88, Q^2^ = 0.42, CV-ANOVA p-value 5.6E-4) and in Fig. 4C the resulting phenotype distinguishing factors are shown. Similarly to what was observed via univariate data analysis, bisected and afucosylated forms (negatively correlating with Jo1^+^ patients) ranked as high contributing factors in distinguishing Jo1^+^ patients from Jo1^−^ patients. Other prominent (Jo1^+^ positively correlating) factors were clinical information such as ASS, ILD, MSA and higher MMT8 as well as the agalactosylation status factor (log[FA2_1/FA2_2]). Noteworthy, of the top ten positively Jo1^+^ correlating factors several were proteins (lysozyme, thrombospondin, plasminogen, CD5 antigen like protein and IgD). Additionally, the conserved lambda chain 2, one kappa variable chain, complement factor H related protein and platelet factor 4 correlated with Jo1^+^ status. For more details, see Supplementary Table 6. In Fig. 4B, the cross validated patient scores (circles) are shown for the model. From the scores (“Known”, Fig. 4B) it is evident that the combined factors listed above robustly can distinguish the Jo1^+^ and Jo1^−^ patient groups (p = 4.2E-6). To validate the model further, we used the remaining patient data from other measured patient time points (0.5–14 years from time point 1), and treated these patient profiles as unknowns. The validation cohort (“Predicted”, Fig. 4B) could also assign a majority of the patients to the correct phenotype (p = 8.5E-4).

## Discussion

This study confirms that the Fc-glycan profile of IgG_1_ in ASS/IIM patients contain less galactosylated epitopes compared to healthy controls and further shows that the Fc-glycan profile of Jo1^+^ patients contains less bisected and afucosylated glycans compared to Jo1^−^ patients. Importantly, the Fc-glycan profile that signified Jo1^+^ patients (i.e. a profile with more agalactosylated and less afucosylated and bisected epitopes) were even more pronounced in anti-Jo1 specific enriched IgG.

It is well known that Fc-glycans contain less galactosylated epitopes in other autoimmune disorder and that these correlate with disease activity and severity^19,20^. Furthermore, a recent study has connected Fc-glycosylation regulation with the axis of IL-23 and T_H_17-cell activity and suggest that this is a determining factor for autoimmune disease onset^21^. The Fc-galactosylation status of IgG can regulate binding to complement 1q factor and complement-dependent toxicity (CDC), thereby influencing the inflammatory status. Furthermore, sialylated Fc-glycans (which can only be attached to galactosylated epitopes), have been directly linked to anti-inflammatory activation via SIGN receptor interactions, and reduce CDC efficacy^22,23^.

Since the significantly higher abundance of agalactosylated FA2 (p = 4.5E-4) in ASS/IIM patients is IgG_1_ specific we tested if the Fc-galactosylation status of IgG_2_ could serve as an internal control used to intra-individually correct for other factors that are also know to affect the galactosylation status of the N-glycan profile such as age, gender and heritability^18,24^. Hence, by using the factor log(FA2_1/FA2_2), ASS/IIM and controls were distinguished with an AUC of 79 ± 6%. This is an improvement to other Fc-galactosylation status measuring factors such as log(FA2/FA2G2) of IgG_1_, which on this data generated an AUC of 71 ± 6% and log(FA2/(FA2G1 + FA2G2) of IgG_1_ which on this data gave an AUC of 73 ± 6%, respectively^10,25^. From investigating the patients with most sampling time points, it was also evident that the log(FA2_1/FA2_2) factor is more stable than log(FA2/FA2G2), Supplemental Fig. 1B. However, whether the log(FA2_1/FA2_2) factor is more specific for IIM than for RA needs to be evaluated further. For example, in contrast to ASS/IIM patients, the galactosylation status of both IgG_1_ and of IgG_2_ have been shown to be skewed in RA^10^. Noteworthy, both the subgroup of Jo1^+^ patients and the subgroup of patients diagnosed with ILD/ASS further improved the sensitivity of the analysis (AUC of 88 ± 6%, Fig. 2B). The majority of ILD/ASS diagnosed patients were also Jo1^+^ (Table 1). However, 10 of 28 patients included in the ILD/ASS group were Jo1^−^ patients seropositive for other aaRS. Hence, the over-representation of Fc-agalactosylated IgG may suggest a lung-tailored bias, as virtually no other IIM extra-muscular manifestations were associated with this glycan feature.

Our hypothesis is that the agalactosylated features of ASS/IIM IgG (and anti-Jo1-IgG) are enhanced due to underlying lung disease related mechanisms in these patients. This hypothesis is in line with a growing body of research that indicate a possible immunological role of IgG Fc-glycans in the respiratory tract. For example, the Fc-galactosylation in chronic lung disease (sarcoidosis and severe asthma) is skewed^26^ and lung cancer patients display a high degree of Fc-agalactosylation which is inversely correlated with good diagnostic performance^27^. Furthermore, note that RA often is affecting the lungs^28^.

In contrast to the galactosylation status that was more prominently skewed both in Jo1^+^ and in the ASS/ILD subgroup, the lower abundance of bisected- and core afucosylated Fc-glycans is more Jo1^+^ phenotype explicit. This hypothesis was further strengthened by analysis of Jo1 specific IgG which contained even lower abundance of bisected- and core afucosylated Fc-glycans than total IgG from the same patients. The profiling of IgG Fc-glycans of specific disease autoantibody reactivities is well characterized in ACPA from RA patients^15^. Noteworthy, anti-Jo1-IgG and ACPA Fc-glycan profiles have several similarities. In addition to higher content of agalactosylated epitopes ACPA-IgG_1_ contains a lower abundance of afucosylated and bisected forms. This might indicate that IgG-Fc-regulatory properties of ACPA and anti-Jo1 are similar and potentially consistent with other autoantibodies. Both bisected- and core afucosylated Fc-glycans increase IgG affinity to FcγRII and III receptors, potentially resulting in more potent antibody dependent cell-mediated cytotoxicity (ADCC)^29,30^. Thus, lower abundance of these epitopes might indicate an inverse effect.

To further evaluate and test how the anti-Jo1^+^ Fc-glycan profile correlate with clinical manifestations and proteomic information of IgG chain distributions and trace proteins (measured in the IgG enriched samples), MVA-analyses were performed. As expected (and in line with the hypothesis that the anti-Jo1 Fc-glycan characteristic features are inflammatory regulating), several of the trace proteins found to positively correlate with the Jo1^+^ patients are involved in inflammatory processes (complement-related, lysozyme and IgD, Fig. 4C). In addition, clinical variables such as ASS and ILD, and proteins (plasminogen and thrombospondin) which have previously been linked to myositis were positively correlating with the Jo1^+^ patients^31–34^. Noteworthy, both the log(FA2_1/FA2_2) and the log Σ[B] data correlated weakly but significantly (R^2^ = 0.13, p = 0.003 and R^2^ = 0.09, p = 0.01, respectively) with accessible C reactive Protein (CRP) data linked to 70 of the measuring time points, (from 23 of the Jo1^−^ and of the 17 Jo1^+^ patients). However, in contrast to the Fc-glycan profile, the CRP data was not significant between the two ASS/IIM phenotypes (p = 0.8), thus indicating that the Fc-glycan profile give more precise information on the underlying immunological patient status.

In conclusion, our study shows that both total IgG and specifically anti-Jo1-IgG from ASS/IIM patients display IgG Fc-glycans that comprise features associated with pro-inflammation (i.e. agalactosylation), which were also overrepresented in patients diagnosed with ILD/ASS. Furthermore, the lower abundance in bisected and afucosylated forms appears to be directly linked to anti-Jo1 autoimmune IgG. Functional studies with glycan manipulation recurring to anti-Jo1-IgG (and other autoantibodies) will shed insights into the role of these Fc-glycan aberrations in anti-Jo1 autoantibodies.

## Methods

### Patients

IIM patients (n = 44) fulfilled the Bohan and Peter^35,36^ criteria for definite, probable or possible polymyositis (PM) or dermatomyositis (DM). Inclusion Body Myositis (IBM) was diagnosed according to Griggs criteria^37^. Because samples were collected between 1996 and 2016, the new EULAR/ACR classification criteria for adult and juvenile IIM had not been approved and therefore the old classification criteria was applied^38^. The diagnosis of ASS was based on the presence of aaRS antibodies, plus one of the following features: ILD, myositis, arthritis, Raynaud’s phenomenon, fever, or mechanic hands^39^. Clinical characteristics of ASS/IIM patients are summarized in Table 2 and Supplementary Table 2. The study was approved by the Ethics Committee at Karolinska Institutet, Karolinska University Hospital, and informed consent was obtained from all participating subjects. All experiments were performed in accordance with relevant guidelines and regulations of Karolinska Institutet and Karolinska University Hospital.

### Antibody purification

Total IgG from serum (0.35–1.5 mL) of 44 ASS/IIM myositis patients, Jo1^+^ n = 19; Jo1^−^ n = 25, and 24 age/sex-matched healthy controls (HC) was isolated as previously described^40^. Longitudinal sera samples were available from eleven Jo1^+^ (i.e. T1–T10) and seven Jo1^−^ (i.e. T1–T2) patients, (Supplemental Table 1). Anti-Jo1-IgG was enriched after pooling IgG from eight of the Jo1^+^ patients sampled at time points T1–T10^40^. From three Jo1^+^ patients a larger volume of sample (9–21 mL) was available at one time point for isolation of anti-Jo1-IgG. For details, see Supplemental Fig. 2A, Supplemental Table 1, Supplemental Table 2, and Supplemental methods.

### IgG sample preparation and LC-MS/MS analysis

Samples were digested with trypsin in duplicates according to previously described protocols^15,25^. Briefly, ten µg of IgG/sample were dissolved to a final volume of 70 µL with a final concentration of 50 mM ammonium bicarbonate (pH 8). The protein was reduced (5 mM dithiothreitol, 30 min, 56 °C) and alkylated (14 mM iodoacetamide, 30 min in darkness). Trypsin was added in a ratio of 1:50 (enzyme:protein) for overnight digestion at 37 °C. Peptides were then desalted using C18 HyperSep Filer Plates (Thermo Fisher Scientific, Waltham, MA). For peptide elution, 60% acetonitrile in 0.1% formic acid was used. Samples were dried using SpeedVac and stored at −20 °C until LC-MS/MS analysis. Samples were randomized both during the sample work up and during the LC-MS/MS analyses. One microgram of digest/sample was analysed using an UltiMate 3000 system connected to an Elite Orbitrap mass spectrometer (both Thermo Fisher Scientific). Reversed phase nano-LC-separation of the peptides was performed on a 15 cm long EASY spray column (PepMap, C18, 2 µm, 100 Å). LC-gradient and MS conditions are described in the supplementary information. Information on run-to-run variation (obtained from a polyclonal IgG standard run in between samples) and from variation between IgG digests are given in Supplemental Table 7 and in Supplemental Fig. 5, respectively.

### Fc-glycopeptide analysis

Using in-house developed software (Quanti)^41^, and as previously described^15,25^, glycopeptide ion abundances were integrated and quantified in a label free manner. The program was set to detect and quantify doubly and triply charged ions from Fc-glycopeptides (IgG_1_:EEQ**Y**NST**Y**R, IgG_2_ or IgG_3_: EEQ**F**NST**F**R and IgG_4_ or IgG_3_: EEQ**F**NST**Y**R/EEQ**Y**NST**F**R) as well as triply and quadruply charged ions from (IgG_1_:TKPREEQ**Y**NST**Y**R, IgG_2_ or IgG_3_: TKPREEQ**F**NST**F**R and IgG_4_ or IgG_3_: TKPREEQ**F**NST**Y**R/TKPREEQ**Y**NST**F**R) using their accurate monoisotopic masses (within <10 ppm from the theoretical mass values) and within a ± 2 min interval around expected retention times. Note that IgG_3_ occurs as both EEQ**F**NST**F**R and EEQ**Y**NST**F**R, with EEQ**Y**NST**F**R being less frequent in Caucasians (which were predominant in this study)^15,42,43^. In total 63 glycopeptide variants were screened for (i.e. 21 glycoforms substituting the three Fc-peptide variants, Supplemental Table 8). Examples of extracted ion chromatograms of the Fc-glycopeptides from EEQ**Y**NST**Y**R (IgG_1_) are shown in Supplementary Fig. 6 from one of the healthy controls and one of the Jo1 specific IgG samples, respectively. For each sample, glycoform abundances were normalized to total content (100%) of Fc-glycosylated IgG_1_, total content (100%) of Fc-glycosylated IgG_2/(3)_ and total content (100%) of Fc-glycosylated IgG_4/(3)_ peptides, respectively. Glycoforms detected at levels constituting less than 0.05% of the total IgG Fc-glycan profiles were discarded. The sub-grouping of respective glycan feature (shown in Table 3) was done by taking the sum of the individual glycopeptide abundances (%) obtained for respective glycopeptide. Note that this was done independent on other features in the profile. Thus, when taking the sum of all galactosylated forms (containing galactose Σ[G]) this was done independent of other glycan substitution patterns, such as N-acetylglucosamine bisection or fucosylation. The log ratio between FA2 of IgG_1_ to FA2 of IgG_2_, (i.e. log(FA2_1/FA2_2) was also performed on the relative distribution values (%) obtained by IgG_1_ and IgG_2_, respectively.

### Proteomics analysis of the IgG enriched samples

In addition to Fc-glycan profiling, samples were simultaneously subjected to regular peptide sequencing of MS/MS spectra^15,25^. For details, see supplemental material.

### Statistics

Univariate analyses on glycopeptides were performed using two tailed Student’s t-test (with equal or unequal variance depending on F-test) or paired t-test. P-values were FDR corrected according total number of comparisons (n = 754). Prism, GraphPad Software was used for ROC-curve and linear regression analysis as well as for analysis of other factors via 1) Wilcoxon matched-pairs signed rank test, 2) Fisher’s exact test or Chi-square test and 3) Kruskal-Wallis or Mann-Whitney tests. Multivariate modelling using Principal Component Analysis (PCA) and Orthogonal projections to latent structures discrimination analysis (OPLS-DA) was performed using SIMCA 15.0. For details, see Supplementary Material.

## Electronic supplementary material

Supplementary information
