# Supplementary material for: Patients with anti-Jo1 antibodies display a characteristic IgG Fc-glycan profile which is further enhanced in anti-Jo1 autoantibodies
Source: Sci Rep. 2018 Dec 18;8:17958. doi: 10.1038/s41598-018-36395-z (PMC6298993; doi:10.1038/s41598-018-36395-z)
Supplement: Supplementary file 1 — Supplementary information [file 41598_2018_36395_MOESM1_ESM.docx]

**Supplementary information**

Patients with anti-Jo1 antibodies display a characteristic IgG Fc-glycan profile which is further enhanced in anti-Jo1 autoantibodies

Cátia Fernandes-Cerqueira, Nuria Renard, Antonella Notarnicola, Edvard Wigren, Susanne Gräslund, Roman A Zubarev, Ingrid E Lundberg, Susanna L Lundström

Supplementary Methods: Page 2 to 6

Supplementary Results: 7

Supplementary Table 1: Page 8

Supplementary Table 2: Page 9

Supplementary Table 3: Page 10

Supplementary Table 4: Page 11

Supplementary Table 5: Page 12-14

Supplementary Table 6: Page 15-19

Supplementary Table 7: Page 20

Supplementary Table 8: Page 21

Supplementary Table 9: Page 22 to 25

Supplementary Figure 1: Page 26

Supplementary Figure 2: Page 27-28

Supplementary Figure 3: Page 29

Supplementary Figure 4: Page 30

Supplementary Figure 5: Page 31

Supplementary Figure 6: Page 32

Supplementary Figure 7: Page 33

References: Page 34

**Supplementary Methods**

### **Production of biotinylated recombinant human Jo1 antigen in *Escherichia coli*:** Human HisRS (amino acids 1-509, Uniprot ID P12081) was cloned into the expression vector pNIC-Bio3 (Genbank acc. no JN792439) which carries an N-terminal His_6_-tag and a C-terminal Avi tag. The recombinant plasmid was transformed into *E. coli* expression strain BL21(DE3) R3 pRARE2 carrying a plasmid for co-expression of BirA ligase. Transformed expression host cells were grown at 37°C to an OD_600_ of 1.5–2.0 in Terrific broth (TB) medium supplemented with Kanamycin (50 µg/mL), Chloramphenicol (35 µg/mL), Spectinomycin (50 ug/mL) and 100 uM Biotin. Temperature was reduced to 18°C and protein expression was induced by addition of IPTG to a final concentration of 0.5 mM. After ~20 hours cells were harvested by centrifugation at 4500 × *g* for 15 minutes and re-suspended in lysis buffer (50 mM HEPES pH 8.0, 500 mM NaCl, 5% glycerol, 10 mM imidazole, 0.5 mM TCEP and Complete EDTA-free protease inhibitor) and stored at –80°C. After thawing, the re-suspended cells were sonicated on ice followed by centrifugation at 44000 × *g* for 50 minutes. The soluble fraction was decanted, filtered (0.45 um) and subsequently loaded onto a HiTrap Ni-chelating column (GE Healthcare) on an ÄKTA Xpress (GE Healthcare). After washing with 20 mM HEPES pH 7.5, 500 mM NaCl, 5% glycerol, 10 mM imidazole and 0.5 mM TCEP, the protein was eluted in 20 mM HEPES pH 7.5, 500 mM NaCl, 5% glycerol, 250 mM imidazole and 0.5 mM TCEP. The eluate was applied to a Hiload XK16/60 Superdex 200 column (GE Healthcare) equilibrated with 20 mM HEPES, pH 7.5, 300 mM NaCl, and 0.5 mM TCEP. Relevant fractions were pooled and analyzed by SDS-PAGE and mass spectrometry. The final protein batch was then flash frozen in liquid nitrogen and stored at -80°C until use.

**Preparation of Jo1 affinity column for isolation of anti-Jo1 IgG:** To prepare the Jo1 affinity column 1 mg of recombinant Jo1 (non-biotinylated) was coupled to N-hydroxysuccinimide activated pre-packed sepharose column (size 1 mL, Ref 17071601 GE Healthcare) according to manufacturer instructions.

**Anti-Jo1 IgG ELISA:** For detection of anti-Jo1 IgG, 96 well plates were coated overnight at 4°C with 50 ng of streptavidin (Sigma) diluted in PBS. The day after, plates were washed 3 times with PBS 0.05% Tween (PBST), blocked with PBST 0.1% BSA (blocking buffer, 100 µL/well) 1hour at room temperature (RT), and incubated with recombinant biotinylated full length Jo1 diluted in blocking buffer (1 µg/well) for 1hour at RT. Plates were washed as described above and incubated for 90 minutes with serial dilutions of anti-Jo1 IgG or corresponding FT fraction (ranging from 0.05 ng/mL up to 1000 ng/mL per well in duplicates). Antibodies added to the plate were diluted in buffer containing 1% BSA, 350 mM NaCl, 10 mM Tris-HCl (pH 7.6), 1% Triton X-100 and 0.1% SDS (RIA buffer). Thereafter, plates were washed and goat anti-human IgG HRP-conjugated antibody (Ref. 109-036-008 Jackson ImmunoResearch) diluted 1:10 000 in RIA buffer (50 µL/well) was added for 1h at RT. Following one last wash step, the absorbance at 450 nm was recorded after 15 minutes incubation with 3,3’,5,5’-Tetramethylbenzidine substrate (reaction was stopped with 1M H_2_SO_4_).

**SDS-PAGE:** Samples derived from the IgG purification protocol (serum, serum-depleted IgG=protein G flow through, PGFT, total IgG, non-Jo1 reactive IgG (Jo1FT) and anti-Jo1 IgG) were diluted in lithium dodecyl sulfate (LDS) sample buffer (Thermo Fisher Scientific) containing 500 mM dithiothreitol (DTT). After denaturation and reduction at 70°C for 10 minutes proteins were loaded onto a NuPAGE® Bis-Tris 4%-12% gel (Thermo) and run in 2-(N-morpholino) ethanesulfonic acid (MES)- sodium dodecyl sulfate (SDS) antioxidant-containing running buffer at 200 V for 40-45 minutes. Protein bands were visualized by Coomassie brilliant blue staining (0.2% Coomassie Brilliant Blue, 7.5% glacial acetic acid, 50% methanol).

**Dot-blot:** Recombinant (r)Jo1 (1µg) was directly spotted on a nitrocellulose membrane and let dry for ~15 minutes. Membrane-containing rJo1 was blocked with 5% milk/PBS 0.1% Tween 20 (PBST) for 1hour at RT and incubated overnight at 4^o^C with serum (1:100), serum-depleted IgG (PGFT 100 or 1 000 ng/mL), total IgG (100 or 1 000 ng/mL), Jo1FT (100 or 1 000 ng/mL), and anti-Jo1 IgG (100 or 1 000 ng/mL). After washing three times with PBST, membranes were probed for 1hour at RT with anti-human IgG diluted 1:10 000 (Ref. sc-2769 Santa Cruz Biotechnology). As a positive control, membrane-containing rJo1 was incubated overnight at 4^o^C with commercial anti-Jo1 antibody (Ref: ab-77034 Abcam) diluted 1:2 000 and probed for 1h at RT with secondary anti-mouse antibody diluted 1:1 000 (Ref: P0447 Dako Agilent). All membranes were washed three times with PBST and developed using SuperSignal® West Pico Chemiluminescent Substrate kit (Thermo).

**LC-MS/MS analysis:** Samples were re-suspended in 0.1% formic acid and 0.5% acetonitrile solution and injected on the column in 5 µL aliquots containing ~1.0 µg of digest. An UltiMate 3000 system connected in-line to an Elite Orbitrap mass spectrometer (both - ThermoFisher Scientific) was used. Reversed phase nano-LC-separation of the peptides was performed on a 15 cm long EASY spray column (PepMap, C18, 2 µm, 100 Å). The chromatographic separation was achieved using a gradient solvent system containing (A) water with 2% acetonitrile and 0.1% formic acid and (B) acetonitrile with 2% water and 0.1% formic acid. The gradient was set up as follows: 3% (B) in 5 min, 3-26% (B) in 115 min, 26−40% (B) in 14 min, 40-95% (B) for 2 min 95% (B) for 6 min and 95-3% (B) for 2 min. The flow rate was set at 300 nL/min. The mass spectrometer was operating in positive ion mode. A survey mass spectrum was acquired in the range of m/z 350-2000 with a nominal resolution of 60,000. Following each MS scan, top ten most abundant precursor ions were selected for MS/MS with HCD for quantitative analysis on individual samples. For qualitative glycopeptide validation ETD and CID fragmentation were used on pooled samples.

**Proteomics analysis of the IgG enriched samples:** The MS/MS information were extracted from.raw files into.mgf files using in-house written RAW_to_MGF software.^1^ Mascot (Matrix Science) search engine v.2.3.02 was used for protein identification with a concatenated version of the SwissProt protein sequence database (Human, 40,548 sequences). Peptide mass error tolerance was set at 10 ppm, MS/MS fragment mass accuracy at 0.05 Da and tryptic digestion was set with a maximum of two missed cleavages. Carbamidomethylation of cysteine was used as a fixed modification, while the variable modifications were asparagine and glutamine deamidation and methionine oxidation. Peptide and protein quantification was performed with in-house written software Quanti.^1^

**Statistics:** Univariate analyses on glycopeptides were performed using two tailed Student’s t-test (with equal or unequal variance depending on F-test) or paired t-test (for matched FT, pools and anti-Jo1 enriched samples). P-values were FDR corrected according total number of comparisons (n=754) with p<5.0E-3 remaining significant following the correction. Comparison of categorical variables were performed using Fisher’s exact test (t-tailed p-values), or Chi-square test when appropriate. Kruskal-Wallis or Mann-Whitney tests were employed when quantitative variables were compared among all groups or between anti-Jo1^+^ and anti-Jo1^-^ groups, respectively. Wilcoxon matched-pairs signed rank test was used when comparing values from samples taken at longitudinal time points. Multivariate modelling using Orthogonal projections to latent structures discrimination analysis (OPLS-DA) was performed using SIMCA 14.0 following mean centring, log transformation and UV scaling. Model performance was reported as cumulative correlation coefficients for the model (R2X[cum]), predictive performance based on seven-fold cross validation calculations (Q2[cum]) and *p*[CV-ANOVA]).

**Patient samples collected at different time points for anti-Jo1 IgG purification:** From 11 Jo1^+^ patients we could retrieve several sera samples from the myositis biobank at Karolinska University Hospital. Those longitudinal sera (Supplementary Table 1) were tested by ELISA to confirm that all were Jo1 reactive, before proceeding with the total IgG enrichment. Sera from 8 out of the 11 patients were still Jo1 seropositive at the different longitudinal time points (Supplementary Table 1). Total IgG was isolated from the sera of the 11 patients and samples were processed for glycan analysis. Further, total IgG belonging to the 8 Jo1^+^ patients from whom longitudinal sera had tested positive in the anti-Jo1 ELISA were pooled in order to have a larger amount of IgG available for anti-Jo1 IgG purification^2^. Since we previously demonstrated the percentage of RA-ACPA in circulation to be between 1-2% of total IgGs, we estimated anti-Jo1 IgG to fall in a similar percentage.

Because longitudinal samples of total IgG isolated from Jo1^+^ sera (n=11) were available, we further isolated total IgG from Jo1^-^ sera (n=7) available from two time points, so we could compare the total IgG glycan profile from the Jo1^+^ and Jo1^-^ patients at different disease time points.

From 11 Jo1^+^ patients, sera were available from 2 time points; from 5 Jo1^+^ patients sera were available from 3 time points; from 3 different Jo1^+^ patients, sera were available from 7, 8 and 10 time points (Supplementary Table 1; Supplementary Table 2).

**Supplementary Results**

*MVA correlation analysis according to ASS/IIM vs controls:* A robust OPLS-DA model (one predictive and one orthogonal component, R^2^=0.75, Q^2^=0.37, CV-ANOVA p-value 7.7E-6) was obtained when investigating and ranking factors (glycans, serum proteins from simultaneous proteomics analysis of samples as well as sex and age) that most prominently correlated with IIM. For details see Supplementary Table 9. In Supplementary Figure 7A, the tCV scores (circles) of the patients and controls are shown and in Figure 7B factors that correlated positively or negatively with 95% confidence with ASS/IIM are shown. Log[FA2_1/FA2_2) was the factor that ranked highest in correlating with ASS/IIM. Overall the sum of agalatosylated IgG_1_glycans Σ[aG_1] and the sum of agalatosylated IgG glycans Σ[aG_total] correlated strongly with ASS/IIM. Inversely, galactosylated factors Σ[G_1] and Σ[G_total] correlated strongly with controls (i.e. negatively with ASS/IIM). In addition to Fc-agalactosylated glycans, the relative abundance of low affinity binding Fc-receptor III-A, IgG_1_ and three specific variable chains (one lambda, one kappa and one heavy chain) correlated with ASS/IIM. To validate the IIM distinguishing model further, we used additional data obtained from the same ASS/IIM patients but from other sampling time points (0.5-14 years from timepoint 1, T1). This validation cohort (treated as unknown patients, predicted scores: tPS, diamonds, Figure 7A) assigned the majority of patients to the ASS/IIM subgroup and remained significantly different compared to controls (p=7.6E-6).

**Supplementary Table 1.** Longitudinal patient sera samples available for IgG enrichment. Note that the samples were collected retrospectively at available time points during disease follow up. The decreasing number of samples is not due to disease free survival, mortality of study fall out

|  |  | **Availability of longitudinal samples** | | | | | | | | | |
| --- | --- | --- | --- | --- | --- | --- | --- | --- | --- | --- | --- |
| **Patient ID** | **Jo1 status** | **T1** | **T2** | **T3** | **T4** | **T5** | **T6** | **T7** | **T8** | **T9** | **T10** |
| P1 | Negative | 0 | 12 |  |  |  |  |  |  |  |  |
| P2 | Negative | 0 | 12 |  |  |  |  |  |  |  |  |
| P3 | Negative | 0 | 7 |  |  |  |  |  |  |  |  |
| P4 | Negative | 0 | 12 |  |  |  |  |  |  |  |  |
| P5 | Negative | 0 | 5 |  |  |  |  |  |  |  |  |
| P6 | Negative | 0 | 7 |  |  |  |  |  |  |  |  |
| P7 | Negative | 0 | 14 |  |  |  |  |  |  |  |  |
| P8 | Negative | 0 |  |  |  |  |  |  |  |  |  |
| P9 | Negative | 0 |  |  |  |  |  |  |  |  |  |
| P10 | Negative | 0 |  |  |  |  |  |  |  |  |  |
| P11 | Negative | 0 |  |  |  |  |  |  |  |  |  |
| P12 | Negative | 0 |  |  |  |  |  |  |  |  |  |
| P13 | Negative | 0 |  |  |  |  |  |  |  |  |  |
| P14 | Negative | 0 |  |  |  |  |  |  |  |  |  |
| P15 | Negative | 0 |  |  |  |  |  |  |  |  |  |
| P16 | Negative | 0 |  |  |  |  |  |  |  |  |  |
| P17 | Negative | 0 |  |  |  |  |  |  |  |  |  |
| P18 | Negative | 0 |  |  |  |  |  |  |  |  |  |
| P19 | Negative | 0 |  |  |  |  |  |  |  |  |  |
| P20 | Negative | 0 |  |  |  |  |  |  |  |  |  |
| P21 | Negative | 0 |  |  |  |  |  |  |  |  |  |
| P22 | Negative | 0 |  |  |  |  |  |  |  |  |  |
| P23 | Negative | 0 |  |  |  |  |  |  |  |  |  |
| P24 | Negative | 0 |  |  |  |  |  |  |  |  |  |
| P25 | Negative | 0 |  |  |  |  |  |  |  |  |  |
| P26 | Positive | 0 | 8 | 53 | 67 | 108 | 121 | 134 | 142 | 165 | 177 |
| P27 | Positive | 0 | 8 | 19 | 25 | 34 | 87 | 98 | 123 |  |  |
| P28 | Positive | 0 | 7 | 13 | 33 | 45 | 58 | 68 |  |  |  |
| P29 | Positive | 0 | 9 | 21 |  |  |  |  |  |  |  |
| P30 | Positive | 0 | 5 | 11 |  |  |  |  |  |  |  |
| P31 | Positive | 0 | 6 |  |  |  |  |  |  |  |  |
| P32 | Positive | 0 | 5 |  |  |  |  |  |  |  |  |
| P33 | Positive | 0 | 17 |  |  |  |  |  |  |  |  |
| P34 | Positive | 0 | 6 |  |  |  |  |  |  |  |  |
| P35 | Positive | 0 | 33 |  |  |  |  |  |  |  |  |
| P36 | Positive | 0 | 13 |  |  |  |  |  |  |  |  |
| P37 | Positive | 0 |  |  |  |  |  |  |  |  |  |
| P38 | Positive | 0 |  |  |  |  |  |  |  |  |  |
| P39 | Positive | 0 |  |  |  |  |  |  |  |  |  |
| P40 | Positive | 0 |  |  |  |  |  |  |  |  |  |
| P41 | Positive | 0 |  |  |  |  |  |  |  |  |  |
| P42* | Positive | 0 |  |  |  |  |  |  |  |  |  |
| P43* | Positive | 0 |  |  |  |  |  |  |  |  |  |
| P44* | Positive | 0 |  |  |  |  |  |  |  |  |  |

P – Patient; T - Time point; 0 – First available sample; T2-T10 – Time points identified by the number of months from T1; Patients with large volume of sample available.

**Supplementary Table 2** Subjects demographics at follow-up

|  | **Anti-Jo1^-^** | | **Anti-Jo1^+^** | | | | | | | | | |
| --- | --- | --- | --- | --- | --- | --- | --- | --- | --- | --- | --- | --- |
|  | **Time 1 (n=7)** | **Time 2 (n=7)** | **Time 1 (n=11)** | **Time 2 (n=11)** | **Time 3 (n=5)** | **Time 4 (n=3)** | **Time 5 (n=3)** | **Time 6 (n=3)** | **Time 7 (n=3)** | **Time 8 (n=2)** | **Time 9 (n=1)** | **Time 10 (n=1)** |
| **Age**, mean years (SD) | 62.4 (11.2) | 63.1 (11.2) | 54.0 (12.0) | 54.9 (11.8) | 52.4 (11.1) | 47.0 (11.0) | 49.0 (11.4) | 51.0 (10-0) | 52.0 (10.0) | 48.5 (8.5) | 59.0 | 60.0 |
| **Disease duration** in months, median (25-75th percentiles) | 24  (0-96) | 31  (12-108) | 36  (12-60) | 45  (17-69) | 57  (18-78) | 73  (33-103) | 82  (45-144) | 135  (58-157) | 146  (68-178) | 175  (171-178) | 201 | 213 |
| **CK**, median µcat/L  (25-75^th^ percentiles) | 7.4  (2.5-27) | 2.05  (1.4-2.9) | 1.3  (0.9-21.1) | 1.6  (0.86-2.0) | 1.5  (0.9-2.8) | 1.6  (0.5-2.7) | 2.2  (0.8-3.6) | 0.7  (0.7-3.7) | 1.0  (0.6-2.9) | 1.1  (0.4-1.8) | 1.1 | 1.0 |
| **Physician VAS**, median  (25-75^th^ percentiles) | 35  (10-54) | 10  (5-20) | 39  (0-56) | 5  (0-16) | 11  (3-14) | 5  (4-5) | 2  (0-4) | 20  (0-33) | 6  (0-60) | 0 | 5 | 0 |
| **Patient VAS**, median  (25-75^th^ percentiles) | 40  (8-51) | 10  (0-49) | 15  (5-78) | 4  (0-55) | 23  (15-43) | 37  (4-70) | 41  (10-72) | 16  (14-48) | 37  (5-57) | 37  (9-64) | 26 | 41 |
| **MDAAT**, median  (25-75^th^ percentiles) | 0.05  (0-0.08) | 0.01  (0-0.10) | 0.122  (0-0.16) | 0.014  (0-0.07) | 0.013  (0-0.07) | 0.025 | 0.002 | 0.010 | 0.015 | 0.00 | 0.023 | 0.00 |
| **HAQ** (1–3), median  (25-75^th^ percentiles) | 1.25  (0-1.38) | 0.38  (0-0.88) | 0.63  (0.10-0.94) | 0.13  (0-1.13) | 0.38  (0.25-1.25) | 0.51  (0.38-0.63) | 0.82  (0.5-1.13 | 0.63  (0.13-1.13) | 0.75  (0.13-1.25) | 0.57  (0.13-1) | NA | 1.38 |
| **MMT-8** (0–80), median  (25-75^th^ percentiles) | 73  (65-80) | 78  (74-80) | 80  (78-80) | 80  (79-80) | 80  (77-80) | 80  (79-80) | 78  (76-80) | 79  (76-80) | 80  (73-80) | 75 | 80 | 79 |
| **Immunosuppressive treatment**, n* |  |  |  |  |  |  |  |  |  |  |  |  |
| No treatment | 1 | 0 | 2 | 1 | 0 | 0 | 0 | 0 | 0 | 0 | 0 | 0 |
| 1 treatment | 1 | 1 | 2 | 2 | 0 | 0 | 0 | 0 | 0 | 0 | 0 | 0 |
| 2 or 3 concomitant treatments | 5 | 6 | 5 | 6 | 4 | 2 | 2 | 3 | 3 | 2 | 1 | 1 |
| **Months from Time 1 (T1), median****  (25-75th percentiles) |  | 12  (7-12) |  | 8  (6-13) | 19  (12-37) | 33  (25-67) | 45  (34-108) | 87  (58-121) | 98  (68-134) | 133  (123-142) | 165 | 177 |

VAS physician, physician’s global disease activity assessment; VAS patient, patient’s global disease activity assessment; MDDAT, Myositis Disease Activity Assessment Tool for extramuscular global assessment; HAQ, Health Assessment Questionnaire; MMT-8, Manual Muscle Testing; *1 treatment designates one of the following treatments alone: methotrexate, glucocorticoids, intravenous Ig, abatacept or azathioprine (AZA); 2 or 3 concomitant treatments designate all the possible following combinations: glucocorticoids+AZA, glucocorticoids+cyclophosphamide, glucocorticoids+methotrexate, glucocorticoids+mycophenolate mofetil; glucocorticoids+rituximab, glucocorticoids+cyclophosphamide+ rituximab, glucocorticoids+ methotrexate+ rituximab, glucocorticoids+mycophenolate Mofetil+rituximab. ** Patients at time point 1 are the same at time point 2-10, both for the anti-Jo1^-^ and anti-Jo1^+^ groups (additional description in supplementary methods **-** Patient samples collected at different time points for anti-Jo1 IgG purification).

**Supplementary Table 3** Concentration and proportion of total IgG and anti-Jo1 IgG in serum

|  | **Total IIM**  **(n=44)** | | **Anti-Jo1^+^**  **(n=19)** | | **Anti-Jo1^-^**  **(n=25)** | | **Healthy controls (n=24)** | |  |
| --- | --- | --- | --- | --- | --- | --- | --- | --- | --- |
| IgG concentration mg/mL, median (range) | 8.3  (2.1-20.2) | | 7.4  (2.1-20.2) | | 8.6  (2.8-16.9) | | 8.4  (4.2-13.2) | |  |
| % IgG of total proteins in serum, median (range)^#^ | 15.1  (4.1-32.0) | | 13.5  (4.1-32.0) | | 16.6  (6.4-29.2) | | 13.3  (7.9-21.1) | |  |
|  | | **Anti-Jo1^+^**  **(n=11)*** | |  | |  | |  | |
| Anti-Jo1-IgG concentration mg/mL, median (range) | | 0.06 (0.01-0.13) | |  | |  | |  | |
| % anti-Jo1-IgG of total proteins in serum, median (range) | | 0.09 (0.01-0.30) | |  | |  | |  | |
| Proportion anti-Jo1-IgG of total IgG %, median (range)^##^ | | 1.58 (0.10-3.62) | |  | |  | |  | |

^#^ % IgG of total proteins in serum was calculated based on the amount of serum applied to the Protein G column and the amount of total IgG eluted from the Protein G column. ^##^The proportion of anti-Jo1 IgG of total IgG was calculated based on the amount of total IgG loaded into the Jo1 affinity column and the amount of anti-Jo1 IgG eluted from the Jo1 column (Ossipova *et al* 2014). *Eight of the 11 anti-Jo1^+^ serum samples were pooled from samples taken at longitudinal time points and used for anti-Jo1-IgG purification. Comparisons among groups was done using the Kruskal-Wallis test followed by Bonferroni correction.

**Supplementary Table 4** Concentration and proportion of total IgG in serum at follow-up

|  | **Anti-Jo1^-^** | | **Anti-Jo1^+^** | | | | | | | | | | |
| --- | --- | --- | --- | --- | --- | --- | --- | --- | --- | --- | --- | --- | --- |
|  | **Time 1 (n=7)** | **Time 2 (n=7)** | **Time 1 (n=11)** | **Time 2 (n=11)** | **Time 3 (n=5)** | **Time 4 (n=3)** | **Time 5 (n=3)** | **Time 6 (n=3)** | **Time 7 (n=3)** | **Time 8 (n=2)** | **Time 9 (n=1)** | **Time 10 (n=1)** |  |
| IgG concentration mg/mL, median (range) | 5.8  (2.8-13.8) | 7.1  (2.3-9.8) | 7.3  (5.5-20.2) | 6.9  (1.94-17.3) | 7.9  (3.6-20.2) | 12.3  (4.8-15.1) | 6.9  (4.3-17.2) | 6.7  (4.7-16.6) | 6.6  (5.2-16.0) | 11.3  (6.0-16.6) | 5.3 | 5.7 |  |
| % IgG of total proteins in serum, median (range) | 15.8  (7.6-29.2) | 12.7  (4.4-18.8) | 13.5  (8.1-20.0) | 12.7  (7.7-17.5) | 13.8  (10.4-17.0) | 14.4  (8.0-24.8) | 12.9  (6.6-17.3) | 10.6  (9.1-22.5) | 11.2  (10.7-22.5) | 14.8  (7.1-55.6) | 10.1 | 10.6 |  |

**Supplementary material Table 5** Relative distribution of Fc-glycans in respective IgG-isotype. Average±standard deviations are given as well as p-values for group comparisons. P-values <0.005 (5.0E-3, highlighted) remain significant following FDR correction. Glycan abbreviations are described in Figure 1; IgG fractions nomenclature are described in Supplementary Figure 1.

| **IgG** | **Glycan** | **T1** |  |  |  | **Jo1^+^ enrichment** | |  | **Healthy vs** | |  |  |  |  | **IIM** |  |  |  |
| --- | --- | --- | --- | --- | --- | --- | --- | --- | --- | --- | --- | --- | --- | --- | --- | --- | --- | --- |
|  |  |  |  |  |  |  |  |  | IIM |  | Jo1^-^ |  | Jo1^+^ |  | Jo1^-^/Jo1^+^ | | Anti-Jo1 | |
|  |  | HC | IIM | IIM Jo1^-^ | IIM Jo1^+^ | Pool | FT | Anti-Jo1^+^ | T1 | All T | T1 | All T | T1 | All T | T1 | All T | FT | PE |
| 1 | FGlcNAc | 0.3±0.5 | 1±1 | 0.4±0.6 | 1±1 | 0.4±0.4 | 0.4±0.4 | 0.4±0.4 | 4.5E-01 | 9.5E-01 | 6.1E-01 | 7.0E-01 | 3.9E-01 | 8.6E-01 | 6.4E-01 | 4.89E-01 | 5.3E-01 | 5.4E-01 |
|  | A2 | 1±1 | 1±1 | 1±1 | 1±1 | 0.8±0.5 | 0.9±0.5 | 0.4±0.4 | 7.7E-02 | 2.2E-02 | 1.5E-02 | 7.4E-04 | 6.6E-01 | 4.6E-01 | 1.1E-01 | 1.98E-03 | 1.5E-03 | 6.6E-02 |
|  | A2B | 1±1 | 1±1 | 1±0.5 | 1±1 | 1.1±0.4 | 1.1±0.4 | 1.0±0.4 | 1.8E-01 | 1.6E-01 | 1.9E-01 | 4.8E-02 | 3.6E-01 | 4.6E-01 | 8.5E-01 | 7.43E-02 | 6.7E-01 | 5.6E-01 |
|  | A2G1 | 1±1 | 1±1 | 2±1 | 1±1 | 1±1 | 1±1 | 1±1 | 6.4E-01 | 5.2E-01 | 1.4E-01 | 1.5E-02 | 1.6E-01 | 2.0E-01 | 1.9E-02 | 3.68E-04 | 1.1E-03 | 1.2E-01 |
|  | A2G2 | 1±1 | 1±1 | 1±1 | 0.4±0.4 | 0.5±0.3 | 0.6±0.4 | 0.3±0.4 | 7.0E-01 | 7.3E-01 | 4.7E-01 | 1.0E-01 | 2.1E-02 | 1.0E-02 | 1.0E-02 | 8.64E-04 | 2.1E-03 | 1.5E-01 |
|  | FA1 | 0.4±0.1 | 0.6±0.4 | 0.5±0.3 | 0.6±0.5 | 0.6±0.2 | 0.6±0.2 | 0.8±0.3 | 4.4E-03 | 5.4E-04 | 3.8E-02 | 3.3E-02 | 3.6E-02 | 9.5E-04 | 2.7E-01 | 3.09E-01 | 5.2E-02 | 2.8E-02 |
|  | FA1G1 | 0.2±0.1 | 0.2±0.1 | 0.2±0.1 | 0.2±0.1 | 0.2±0.04 | 0.2±0.1 | 0.2±0.1 | 3.4E-01 | 2.1E-01 | 3.4E-01 | 2.1E-01 | 4.7E-01 | 2.9E-01 | 9.7E-01 | 7.22E-01 | 9.5E-01 | 3.9E-01 |
|  | FA1G1S1 | 0.1±0.1 | 0.2±0.2 | 0.2±0.2 | 0.2±0.2 | 0.2±0.1 | 0.2±0.1 | 0.2±0.4 | 3.5E-02 | 1.0E-01 | 1.6E-01 | 2.3E-01 | 5.9E-02 | 7.2E-02 | 5.3E-01 | 7.57E-01 | 4.2E-01 | 4.2E-01 |
|  | FA2 | 18±6 | 25±10 | 23±10 | 28±9 | 28±8 | 27±4 | 34±9 | 4.5E-04 | 5.8E-04 | 3.9E-02 | 3.8E-02 | 6.2E-05 | 1.1E-05 | 7.9E-02 | 3.17E-02 | 1.4E-02 | 9.8E-02 |
|  | FA2G1 | 28±5 | 26±4 | 25±5 | 26±3 | 26±4 | 27±2 | 26±4 | 4.5E-02 | 7.3E-02 | 5.2E-02 | 1.0E-01 | 2.0E-01 | 2.0E-01 | 4.9E-01 | 4.46E-01 | 3.2E-01 | 9.3E-01 |
|  | FA2G2 | 12±4 | 9±4 | 10±4 | 9±4 | 8±3 | 9±2 | 7±2 | 1.1E-02 | 2.8E-03 | 6.5E-02 | 7.1E-02 | 9.7E-03 | 5.4E-03 | 3.6E-01 | 2.31E-01 | 1.8E-02 | 2.6E-01 |
|  | FA2G1S1 | 2±1 | 2±1 | 2±1 | 2±1 | 2±1 | 2±1 | 2±1 | 6.6E-01 | 4.0E-01 | 6.1E-01 | 2.5E-01 | 7.9E-01 | 6.2E-01 | 9.2E-01 | 4.26E-01 | 6.3E-01 | 9.3E-01 |
|  | FA2G2S1 | 13±6 | 10±5 | 10±5 | 9±5 | 8±3 | 7±3 | 7±4 | 2.3E-02 | 2.5E-02 | 9.7E-02 | 5.1E-02 | 3.6E-02 | 6.9E-03 | 4.1E-01 | 6.38E-01 | 6.7E-01 | 6.0E-01 |
|  | FA2B | 8±3 | 10±4 | 10±4 | 10±4 | 11±3 | 10±2 | 10±3 | 2.9E-02 | 6.0E-03 | 5.1E-02 | 8.9E-02 | 6.3E-02 | 1.3E-03 | 9.5E-01 | 3.40E-01 | 8.0E-01 | 2.6E-01 |
|  | FA2BG1 | 12±3 | 11±3 | 12±3 | 10±2 | 11±3 | 11±2 | 8±3 | 1.1E-01 | 1.4E-01 | 6.8E-01 | 8.1E-01 | 4.9E-03 | 2.1E-02 | 2.7E-02 | 4.17E-02 | 5.1E-03 | 1.8E-02 |
|  | FA2BG2 | 2±1 | 1±1 | 1±1 | 1±1 | 1.1±0.5 | 1.2±0.5 | 0.7±0.5 | 3.2E-02 | 1.8E-02 | 3.2E-01 | 4.0E-01 | 1.9E-03 | 8.5E-04 | 3.5E-02 | 2.58E-02 | 1.1E-03 | 2.5E-02 |
|  | FA2BG1S1 | 0.4±0.2 | 0.3±0.2 | 0.4±0.1 | 0.3±0.2 | 0.3±0.2 | 0.3±0.1 | 0.2±0.1 | 1.5E-01 | 4.8E-02 | 3.8E-01 | 1.7E-01 | 1.1E-01 | 9.1E-03 | 2.7E-01 | 1.63E-01 | 4.0E-01 | 3.3E-01 |
|  | FA2BG2S1 | 0.3±0.2 | 0.3±0.2 | 0.3±0.2 | 0.3±0.2 | 0.2±0.1 | 0.2±0.1 | 0.1±0.1 | 4.8E-02 | 4.7E-02 | 2.0E-01 | 1.5E-01 | 4.2E-02 | 7.4E-03 | 2.8E-01 | 2.67E-01 | 2.0E-01 | 1.4E-01 |
| 2 or (3) | FGlcNAc | 0.1±0.2 | 0.2±0.3 | 0.2±0.3 | 0.2±0.3 | 0.2±0.2 | 0.2±0.2 | 0.1±0.1 | 5.4E-01 | 8.4E-01 | 5.3E-01 | 5.8E-01 | 6.8E-01 | 4.1E-01 | 8.3E-01 | 1.20E-01 | 3.8E-01 | 5.9E-01 |
|  | A2 | 0.4±0.3 | 1±1 | 1±1 | 1±1 | 0.6±0.5 | 0.7±0.6 | 0.5±0.3 | 1.2E-02 | 5.8E-04 | 1.4E-02 | 1.6E-04 | 2.5E-01 | 5.4E-02 | 4.3E-01 | 1.16E-02 | 1.2E-01 | 2.6E-01 |
|  | A2B | 0.7±0.4 | 0.7±0.4 | 1±0.4 | 1±0.3 | 0.7±0.4 | 0.7±0.4 | 0.9±0.5 | 6.4E-01 | 6.0E-01 | 3.6E-01 | 1.6E-01 | 7.4E-01 | 7.3E-01 | 2.4E-01 | 2.42E-02 | 3.4E-01 | 2.4E-01 |
|  | FA1 | 0.9±0.2 | 1±1 | 1±0.4 | 1±1 | 1.1±0.4 | 1.2±0.3 | 1.3±0.5 | 1.4E-01 | 5.6E-02 | 5.5E-01 | 6.6E-01 | 1.6E-01 | 2.4E-02 | 2.4E-01 | 5.32E-02 | 1.8E-01 | 2.5E-02 |
|  | FA1G1 | 0.2±0.1 | 0.2±0.1 | 0.2±0.1 | 0.2±0.1 | 0.2±0.04 | 0.2±0.1 | 0.2±0.1 | 9.5E-01 | 7.0E-01 | 6.6E-01 | 2.9E-01 | 6.4E-01 | 1.0E+00 | 4.8E-01 | 2.54E-01 | 6.1E-01 | 1.9E-01 |
|  | FA1G1S1 | 0.2±0.1 | 0.3±0.2 | 0.2±0.2 | 0.3±0.2 | 0.2±0.1 | 0.2±0.2 | 0.4±0.3 | 1.1E-01 | 3.3E-01 | 3.1E-01 | 3.4E-01 | 1.5E-01 | 3.7E-01 | 4.2E-01 | 9.70E-01 | 1.7E-02 | 6.1E-02 |
|  | FA2 | 33±9 | 36±9 | 35±8 | 39±10 | 39±9 | 40±6 | 43±9 | 1.9E-01 | 6.4E-02 | 5.4E-01 | 5.4E-01 | 8.3E-02 | 1.7E-02 | 1.9E-01 | 2.74E-02 | 1.5E-01 | 1.8E-01 |
|  | FA2G1 | 30±3 | 28±4 | 28±4 | 28±4 | 28±5 | 30±4 | 26±6 | 1.6E-01 | 1.3E-01 | 1.8E-01 | 2.5E-01 | 2.5E-01 | 1.0E-01 | 9.5E-01 | 7.29E-01 | 4.3E-03 | 1.3E-01 |
|  | FA2G2 | 10±4 | 9±4 | 9±4 | 8±4 | 8±3 | 8±3 | 7±3 | 2.0E-01 | 5.8E-02 | 4.2E-01 | 2.2E-01 | 1.5E-01 | 4.5E-02 | 4.5E-01 | 4.02E-01 | 2.5E-02 | 1.6E-01 |
|  | FA2G1S1 | 3±2 | 3±1 | 3±1 | 3±1 | 3±1 | 2±1 | 4±1 | 6.3E-01 | 3.3E-01 | 5.6E-01 | 5.7E-01 | 8.7E-01 | 2.8E-01 | 7.1E-01 | 5.51E-01 | 3.2E-03 | 1.7E-01 |
|  | FA2G2S1 | 6±3 | 5±3 | 5±2 | 5±4 | 4±2 | 2±2 | 5±2 | 3.3E-01 | 1.5E-01 | 2.5E-01 | 1.6E-01 | 6.3E-01 | 2.7E-01 | 7.3E-01 | 7.01E-01 | 1.5E-02 | 4.0E-01 |
|  | FA2B | 9±3 | 10±3 | 10±3 | 9±2 | 9±2 | 10±3 | 8±3 | 4.4E-01 | 1.8E-01 | 1.2E-01 | 7.9E-02 | 5.6E-01 | 4.2E-01 | 3.9E-02 | 1.27E-01 | 8.5E-04 | 3.6E-02 |
|  | FA2BG1 | 6±1 | 5±2 | 6±2 | 4±1 | 5±1 | 5±1 | 4±1 | 1.6E-01 | 3.8E-02 | 8.9E-01 | 8.9E-01 | 1.5E-03 | 1.3E-04 | 1.3E-03 | 9.70E-05 | 2.3E-03 | 6.0E-02 |
|  | FA2BG2 | 0.5±0.3 | 0.4±0.2 | 0.4±0.2 | 0.3±0.2 | 0.3±0.2 | 0.3±0.2 | 0.2±0.1 | 4.6E-02 | 2.1E-02 | 3.2E-01 | 1.8E-01 | 1.3E-02 | 4.3E-03 | 4.8E-02 | 1.18E-02 | 3.1E-02 | 9.6E-02 |
|  | FA2BG1S1 | 0.1±0.1 | 0.1±0.04 | 0.1±0.05 | 0.04±0.03 | 0.03±0.03 | 0.02±0.02 | 0.04±0.03 | 5.9E-01 | 1.4E-01 | 9.5E-01 | 9.8E-01 | 2.2E-01 | 4.0E-02 | 2.0E-01 | 1.05E-02 | 6.9E-02 | 5.9E-01 |
|  | FA2BG2S1 | 0.1±0.1 | 0.1±0.1 | 0.1±0.1 | 0.1±0.1 | 0.1±0.05 | 0.04±0.03 | 0.1±0.05 | 1.1E-01 | 3.3E-02 | 3.9E-01 | 1.8E-01 | 5.7E-02 | 9.9E-03 | 1.5E-01 | 3.06E-02 | 3.8E-01 | 2.3E-01 |
| 3 or 4 | FGlcNAc | 0.2±0.4 | 0.2±0.3 | 0.1±0.2 | 0.3±0.4 | 0.2±0.2 | 0.3±0.3 | 0.3±0.5 | 6.6E-01 | 3.5E-01 | 2.9E-01 | 2.4E-01 | 7.1E-01 | 4.2E-01 | 2.0E-01 | 4.57E-01 | 7.4E-01 | 3.6E-01 |
|  | A2B | 0.4±0.4 | 0.4±0.4 | 0.4±0.4 | 0.4±0.5 | 0.3±0.4 | 0.3±0.3 | 0.2±0.2 | 8.7E-01 | 6.4E-01 | 8.0E-01 | 9.4E-01 | 9.9E-01 | 4.1E-01 | 8.2E-01 | 2.94E-01 | 6.6E-01 | 7.2E-01 |
|  | FA1 | 0.4±0.2 | 0.4±0.2 | 0.4±0.3 | 0.4±0.2 | 0.3±0.3 | 0.3±0.2 | 0.2±0.2 | 3.6E-01 | 5.6E-01 | 3.1E-01 | 4.9E-01 | 6.3E-01 | 6.9E-01 | 6.0E-01 | 5.98E-01 | 4.4E-02 | 3.2E-01 |
|  | FA1G1 | 0.1±0.1 | 0.05±0.1 | 0.05±0.1 | 0.05±0.1 | 0.05±0.1 | 0.03±0.04 | 0.03±0.1 | 9.2E-02 | 1.9E-02 | 1.1E-01 | 4.8E-02 | 2.6E-01 | 1.2E-02 | 8.4E-01 | 3.14E-01 | 9.9E-01 | 5.2E-01 |
|  | FA2 | 29±9 | 34±10 | 33±10 | 34±9 | 31±5 | 32±5 | 29±7 | 6.4E-02 | 1.9E-02 | 1.3E-01 | 3.8E-02 | 9.4E-02 | 3.3E-02 | 8.3E-01 | 4.97E-01 | 1.2E-01 | 1.2E-01 |
|  | FA2G1 | 24±4 | 24±4 | 23±4 | 25±4 | 27±4 | 28±4 | 25±4 | 9.7E-01 | 4.3E-01 | 3.9E-01 | 1.6E-01 | 2.1E-01 | 1.0E-02 | 5.0E-02 | 6.18E-06 | 7.8E-02 | 2.5E-02 |
|  | FA2G2 | 9±3 | 8±4 | 8±3 | 8±4 | 9±2 | 9±3 | 7±2 | 2.2E-01 | 1.0E-01 | 1.9E-01 | 4.6E-02 | 4.6E-01 | 2.9E-01 | 7.2E-01 | 1.24E-01 | 1.4E-02 | 1.5E-02 |
|  | FA2G1S1 | 4±1 | 3±1 | 3±1 | 3±1 | 3±1 | 3±1 | 3±1 | 1.7E-01 | 1.2E-01 | 1.8E-01 | 1.2E-01 | 3.5E-01 | 1.9E-01 | 7.8E-01 | 5.59E-01 | 4.8E-01 | 8.5E-01 |
|  | FA2G2S1 | 9±5 | 7±4 | 7±3 | 8±5 | 7±3 | 6±2 | 8±3 | 5.2E-02 | 2.7E-02 | 3.4E-02 | 8.7E-03 | 3.0E-01 | 3.8E-02 | 5.0E-01 | 1.36E-01 | 1.3E-01 | 7.1E-01 |
|  | FA2B | 15±6 | 15±5 | 16±5 | 13±5 | 13±7 | 13±6 | 19±8 | 8.7E-01 | 7.4E-01 | 4.5E-01 | 1.5E-01 | 2.1E-01 | 1.5E-01 | 1.8E-02 | 1.92E-05 | 2.7E-02 | 4.6E-03 |
|  | FA2BG1 | 8±2 | 8±3 | 8±3 | 7±2 | 8±1 | 7±3 | 8±3 | 7.4E-01 | 5.6E-01 | 6.9E-01 | 9.6E-01 | 1.8E-01 | 2.8E-01 | 1.9E-01 | 2.52E-01 | 7.1E-01 | 9.5E-01 |
|  | FA2BG2 | 1±1 | 1±1 | 1±1 | 1±1 | 1±1 | 1±1 | 1±1 | 1.1E-01 | 4.5E-02 | 4.7E-01 | 3.3E-01 | 1.8E-02 | 9.7E-03 | 1.4E-01 | 2.11E-01 | 5.3E-01 | 4.7E-01 |

T, Time; C, Healthy controls; IIM, idiopathic inflammatory myopathies; FT, flow through - non-Jo1 reactive IIM IgG; PE, Total Jo1^+^ IIM IgG prior enrichment

**Supplementary Table 6** Factors included in the MVA model used to extract factors that could distinguish anti-Jo1^+^ and anti-Jo1^-^ patients with 95% confidence. Glycan abbreviations are described in Figure 1 and in Table 3.

| **Correlation** | **Type** | **Variable** | **Uniprot ID** | **pq[1]** | **±95% confidence interval** |
| --- | --- | --- | --- | --- | --- |
| Jo1^+^ correlating | Clinical Information | ASS |  | 0.21 | 0.05 |
|  | Protein | Lysozyme C | LYSC_HUMAN | 0.16 | 0.11 |
|  | Protein | Plasminogen | PLMN_HUMAN | 0.16 | 0.11 |
|  | Protein | Thrombospondin-1 | TSP1_HUMAN | 0.15 | 0.10 |
|  | Clinical Information | MA-ILD |  | 0.15 | 0.09 |
|  | IgG | Ig kappa chain V-I region Mev | KV120_HUMAN | 0.14 | 0.10 |
|  |  |  |  |  |  |
|  | IgD | Ig delta chain C region | IGHD_HUMAN | 0.14 | 0.08 |
|  | Clinical Information | MSA |  | 0.14 | 0.12 |
|  | IgG | Ig lambda-2 chain C regions | LAC2_HUMAN | 0.13 | 0.07 |
|  | Protein | CD5 antigen-like | CD5L_HUMAN | 0.12 | 0.08 |
|  | Protein | Platelet factor 4 | PLF4_HUMAN | 0.12 | 0.05 |
|  | Protein | Complement factor H-related protein 1 | FHR1_HUMAN | 0.10 | 0.10 |
|  | Glycan | log(FA2_1/FA2_2) |  | 0.09 | 0.08 |
|  | Clinical Information | MMT8 |  | 0.08 | 0.03 |
| Jo1^-^ correlating | Glycan | Σ[B_2] |  | -0.17 | 0.11 |
|  | Glycan | Σ[B_total] |  | -0.16 | 0.12 |
|  | Glycan | Σ[B_34] |  | -0.16 | 0.11 |
|  | Protein | Dermcidin | DCD_HUMAN | -0.14 | 0.14 |
|  | Protein | Apolipoprotein C-I | APOC1_HUMAN | -0.13 | 0.09 |
|  | Glycan | Σ[aF_1] |  | -0.13 | 0.10 |
|  | Glycan | Σ[aF_total] |  | -0.12 | 0.10 |
|  | Age | Age |  | -0.09 | 0.09 |
|  | IgG | Ig kappa chain V-I region Scw | KV117_HUMAN | -0.09 | 0.07 |
|  | IgG | Ig heavy chain V-I region HG3 | HV102_HUMAN | -0.08 | 0.08 |
| Other factors | Protein | Complement component C8 beta chain | CO8B_HUMAN | 0.14 | 0.14 |
|  | Protein | Ribonuclease 4 | RNAS4_HUMAN | 0.12 | 0.14 |
|  | Ig3 | Ig gamma-3 chain C region | IGHG3_HUMAN | 0.12 | 0.17 |
|  | Protein | Fibrinogen alpha chain | FIBA_HUMAN | 0.11 | 0.15 |
|  | Protein | Complement C1q subcomponent subunit A | C1QA_HUMAN | 0.11 | 0.12 |
|  | Protein | Neutrophil defensin 3 | DEF3_HUMAN | 0.10 | 0.15 |
|  | Protein | Clusterin | CLUS_HUMAN | 0.09 | 0.13 |
|  | Protein | Complement C1q subcomponent subunit C | C1QC_HUMAN | 0.09 | 0.10 |
|  | IgM | Ig mu chain C region | IGHM_HUMAN | 0.09 | 0.12 |
|  | Clinical Information | Arthritis |  | 0.09 | 0.19 |
|  | Protein | Complement C1q subcomponent subunit B | C1QB_HUMAN | 0.09 | 0.13 |
|  | IgM | Ig mu heavy chain disease protein | MUCB_HUMAN | 0.08 | 0.11 |
|  | Protein | Gelsolin | GELS_HUMAN | 0.08 | 0.11 |
|  | IgG | Ig heavy chain V-III region BUT | HV306_HUMAN | 0.08 | 0.09 |
|  | Protein | Complement C3 | CO3_HUMAN | 0.08 | 0.08 |
|  | Clinical Information | SSA |  | 0.08 | 0.10 |
|  | Protein | Complement C1r subcomponent | C1R_HUMAN | 0.08 | 0.13 |
|  | IgM | Immunoglobulin J chain | IGJ_HUMAN | 0.08 | 0.11 |
|  | Protein | Low affinity immunoglobulin gamma Fc region receptor III-A | FCG3A_HUMAN | 0.07 | 0.13 |
|  | Glycan | Σ[aG_1] |  | 0.07 | 0.08 |
|  | IgG | Immunoglobulin lambda-like polypeptide 5 | IGLL5_HUMAN | 0.07 | 0.08 |
|  | IgG | Ig kappa chain V-III region SIE | KV302_HUMAN | 0.06 | 0.08 |
|  | Protein | Complement component C8 gamma chain | CO8G_HUMAN | 0.06 | 0.16 |
|  | Protein | Vitamin K-dependent protein S | PROS_HUMAN | 0.06 | 0.14 |
|  | IgG | Ig heavy chain V-I region V35 | HV103_HUMAN | 0.06 | 0.12 |
|  | IgG | Ig kappa chain V-I region EU | KV106_HUMAN | 0.06 | 0.14 |
|  | Protein | Complement factor H | CFAH_HUMAN | 0.06 | 0.10 |
|  | Protein | C4b-binding protein alpha chain | C4BPA_HUMAN | 0.06 | 0.14 |
|  | Clinical Information | Male |  | 0.05 | 0.16 |
|  | Clinical Information | MAA |  | 0.05 | 0.06 |
|  | IgG | Ig kappa chain V-III region VG | KV309_HUMAN | 0.05 | 0.10 |
|  | IgG | Immunoglobulin lambda-like polypeptide 1 | IGLL1_HUMAN | 0.05 | 0.13 |
|  | IgG | Ig heavy chain V-II region SESS | HV208_HUMAN | 0.05 | 0.09 |
|  | Protein | Complement C1s subcomponent | C1S_HUMAN | 0.05 | 0.13 |
|  | Protein | Complement C4-A | CO4A_HUMAN | 0.04 | 0.11 |
|  | IgG | Ig kappa chain V-III region GOL | KV307_HUMAN | 0.04 | 0.16 |
|  | IgG | Ig kappa chain V-I region WEA | KV118_HUMAN | 0.04 | 0.18 |
|  | IgG | Ig heavy chain V-III region GAL | HV320_HUMAN | 0.04 | 0.14 |
|  | IgG | Ig heavy chain V-II region WAH | HV206_HUMAN | 0.04 | 0.12 |
|  | IgG | Ig kappa chain V-II region MIL | KV203_HUMAN | 0.04 | 0.15 |
|  | Protein | Complement C2 | CO2_HUMAN | 0.04 | 0.13 |
|  | Glycan | Σ[G_34] |  | 0.04 | 0.13 |
|  | IgA | Ig alpha-1 chain C region | IGHA1_HUMAN | 0.04 | 0.09 |
|  | IgG | Ig heavy chain V-III region BRO | HV305_HUMAN | 0.03 | 0.12 |
|  | Protein | Kininogen-1 | KNG1_HUMAN | 0.03 | 0.12 |
|  | IgG | Ig kappa chain V-I region Lay | KV113_HUMAN | 0.03 | 0.16 |
|  | Protein | Complement factor H-related protein 5 | FHR5_HUMAN | 0.03 | 0.06 |
|  | Glycan | Σ[S_2] |  | 0.03 | 0.15 |
|  | IgG | Ig heavy chain V-III region WAS | HV315_HUMAN | 0.03 | 0.14 |
|  | IgG | Ig lambda chain V-III region SH | LV301_HUMAN | 0.03 | 0.09 |
|  | Protein | Histidine-rich glycoprotein | HRG_HUMAN | 0.03 | 0.07 |
|  | IgA | Ig alpha-2 chain C region | IGHA2_HUMAN | 0.03 | 0.12 |
|  | Protein | Titin | TITIN_HUMAN | 0.03 | 0.11 |
|  | Protein | Apolipoprotein E | APOE_HUMAN | 0.03 | 0.10 |
|  | Protein | C4b-binding protein beta chain | C4BPB_HUMAN | 0.02 | 0.12 |
|  | IgG | Ig lambda chain V-V region DEL | LV501_HUMAN | 0.02 | 0.14 |
|  | IgG | Ig lambda chain V-I region VOR | LV101_HUMAN | 0.02 | 0.16 |
|  | IgG | Ig kappa chain V-IV region Len | KV402_HUMAN | 0.02 | 0.14 |
|  | IgG | Ig kappa chain V-I region HK102 | KV110_HUMAN | 0.02 | 0.14 |
|  | Glycan | Σ[S_34] |  | 0.02 | 0.10 |
|  | IgG | Ig lambda chain V-III region LOI | LV302_HUMAN | 0.02 | 0.08 |
|  | IgG | Ig lambda chain V-II region TRO | LV204_HUMAN | 0.02 | 0.06 |
|  | Protein | Transthyretin | TTHY_HUMAN | 0.02 | 0.13 |
|  | IgG | Ig lambda chain V-I region NEW | LV103_HUMAN | 0.02 | 0.09 |
|  | IgG | Ig kappa chain V-I region Walker | KV123_HUMAN | 0.02 | 0.06 |
|  | IgG | Ig kappa chain V-I region AG | KV101_HUMAN | 0.02 | 0.12 |
|  | IgG | Ig kappa chain V-III region B6 | KV301_HUMAN | 0.02 | 0.13 |
|  | Protein | Vitronectin | VTNC_HUMAN | 0.01 | 0.05 |
|  | Glycan | Σ[aG_2] |  | 0.01 | 0.08 |
|  | Glycan | Σ[aG_total] |  | 0.01 | 0.10 |
|  | Protein | Putative V-set and immunoglobulin domain-containing-like protein IGHV4OR15-8 | IV4F8_HUMAN | 0.01 | 0.09 |
|  | Glycan | Σ[aF_34] |  | 0.01 | 0.08 |
|  | Protein | Plasma protease C1 inhibitor | IC1_HUMAN | 0.01 | 0.10 |
|  | IgG | Ig heavy chain V-III region VH26 | HV303_HUMAN | 0.01 | 0.08 |
|  | Protein | Complement factor I | CFAI_HUMAN | 0.01 | 0.14 |
|  | IgG | Ig kappa chain V-III region IARC/BL41 | KV311_HUMAN | 0.00 | 0.08 |
|  | Protein | Complement C4-B | CO4B_HUMAN | 0.00 | 0.11 |
|  | Protein | Platelet basic protein | CXCL7_HUMAN | 0.00 | 0.09 |
|  | Clinical Information | Pm-Scl |  | 0.00 | 0.01 |
|  | IgG | Ig kappa chain V-I region Kue | KV112_HUMAN | 0.00 | 0.17 |
|  | IgG | Ig lambda chain V-I region BL2 | LV107_HUMAN | 0.00 | 0.11 |
|  | Protein | Complement C1r subcomponent-like protein | C1RL_HUMAN | 0.00 | 0.11 |
|  | Protein | Serotransferrin | TRFE_HUMAN | 0.00 | 0.14 |
|  | Protein | Fibrinogen beta chain | FIBB_HUMAN | 0.00 | 0.11 |
|  | Protein | Inter-alpha-trypsin inhibitor heavy chain H4 | ITIH4_HUMAN | 0.00 | 0.10 |
|  | Glycan | Σ[S_total] |  | 0.00 | 0.12 |
|  | IgG | Ig heavy chain V-III region LAY | HV314_HUMAN | -0.01 | 0.07 |
|  | IgG | Ig kappa chain V-I region BAN | KV122_HUMAN | -0.01 | 0.07 |
|  | IgG | Ig gamma-4 chain C region | IGHG4_HUMAN | -0.01 | 0.08 |
|  | Protein | Properdin | PROP_HUMAN | -0.01 | 0.15 |
|  | Protein | Apolipoprotein A-I | APOA1_HUMAN | -0.01 | 0.12 |
|  | Protein | Protein AMBP | AMBP_HUMAN | -0.01 | 0.12 |
|  | IgG | Ig kappa chain V-I region Roy | KV116_HUMAN | -0.01 | 0.11 |
|  | IgG | Ig heavy chain V-III region JON | HV319_HUMAN | -0.01 | 0.11 |
|  | Protein | Prothrombin | THRB_HUMAN | -0.01 | 0.08 |
|  | Clinical Information | U1 RNP |  | -0.01 | 0.13 |
|  | Protein | Antithrombin-III | ANT3_HUMAN | -0.01 | 0.09 |
|  | Clinical Information | Ever smoker |  | -0.01 | 0.08 |
|  | Protein | Alpha-2-macroglobulin | A2MG_HUMAN | -0.01 | 0.10 |
|  | Protein | Beta-2-glycoprotein 1 | APOH_HUMAN | -0.01 | 0.10 |
|  | IgG | Ig kappa chain V-I region DEE | KV105_HUMAN | -0.02 | 0.09 |
|  | Protein | Haptoglobin-related protein | HPTR_HUMAN | -0.02 | 0.11 |
|  | Clinical Information | Mi-2 |  | -0.02 | 0.14 |
|  | Glycan | Σ[G_total] |  | -0.02 | 0.11 |
|  | Protein | Fibrinogen gamma chain | FIBG_HUMAN | -0.02 | 0.14 |
|  | IgG | Ig heavy chain V-II region ARH-77 | HV209_HUMAN | -0.02 | 0.11 |
|  | IgG | Ig heavy chain V-III region TRO | HV301_HUMAN | -0.02 | 0.15 |
|  | IgG | Ig heavy chain V-III region WEA | HV302_HUMAN | -0.02 | 0.09 |
|  | Protein | Serum albumin | ALBU_HUMAN | -0.02 | 0.08 |
|  | IgG | Ig lambda chain V-VI region EB4 | LV605_HUMAN | -0.02 | 0.11 |
|  | IgG | Ig heavy chain V-III region CAM | HV307_HUMAN | -0.02 | 0.15 |
|  | Clinical Information | Ku |  | -0.02 | 0.07 |
|  | Clinical Information | EJ(+) |  | -0.03 | 0.07 |
|  | Clinical Information | ANA |  | -0.03 | 0.16 |
|  | IgG | Ig kappa chain V-II region FR | KV202_HUMAN | -0.03 | 0.08 |
|  | Glycan | Σ[G_2] |  | -0.03 | 0.11 |
|  | IgG | Ig gamma-2 chain C region | IGHG2_HUMAN | -0.03 | 0.06 |
|  | Protein | Fibronectin | FINC_HUMAN | -0.03 | 0.13 |
|  | IgG | Ig heavy chain V-III region GA | HV308_HUMAN | -0.03 | 0.10 |
|  | Clinical Information | PL7(+) |  | -0.03 | 0.10 |
|  | Clinical Information | Skin (symptom) |  | -0.03 | 0.11 |
|  | IgG | Ig kappa chain C region | IGKC_HUMAN | -0.03 | 0.09 |
|  | Glycan | Σ[S_1] |  | -0.03 | 0.15 |
|  | IgG | Ig lambda chain V region 4A | LV001_HUMAN | -0.03 | 0.06 |
|  | Clinical Information | PL12(+) |  | -0.04 | 0.08 |
|  | Clinical Information | Dysphagia |  | -0.04 | 0.09 |
|  | IgG | Ig lambda chain V-I region WAH | LV106_HUMAN | -0.04 | 0.10 |
|  | Clinical Information | SRP |  | -0.04 | 0.09 |
|  | IgG | Ig heavy chain V-III region KOL | HV311_HUMAN | -0.04 | 0.14 |
|  | Glycan | Σ[aG_34] |  | -0.04 | 0.14 |
|  | Clinical Information | HAQ |  | -0.04 | 0.09 |
|  | IgG | Ig kappa chain V-I region Wes | KV119_HUMAN | -0.04 | 0.15 |
|  | IgG | Ig kappa chain V-III region NG9 | KV303_HUMAN | -0.05 | 0.10 |
|  | Clinical Information | VAS |  | -0.05 | 0.12 |
|  | IgG | Ig gamma-1 chain C region | IGHG1_HUMAN | -0.05 | 0.07 |
|  | Protein | Complement factor B | CFAB_HUMAN | -0.05 | 0.10 |
|  | IgG | Ig kappa chain V-I region HK101 | KV109_HUMAN | -0.05 | 0.08 |
|  | Clinical Information | Female |  | -0.05 | 0.16 |
|  | Protein | Complement C5 | CO5_HUMAN | -0.06 | 0.12 |
|  | Clinical Information | TIF1gamma |  | -0.06 | 0.11 |
|  | IgG | Ig kappa chain V-I region AU OS=Homo sapiens PE=1 SV=1 | KV102_HUMAN | -0.06 | 0.10 |
|  | Glycan | Σ[G_1] |  | -0.06 | 0.09 |
|  | Clinical Information | OJ(+) |  | -0.06 | 0.08 |
|  | IgG | Ig lambda chain V-IV region Hil | LV403_HUMAN | -0.06 | 0.08 |
|  | Protein | Haptoglobin | HPT_HUMAN | -0.06 | 0.15 |
|  | IgG | Ig kappa chain V-I region CAR | KV104_HUMAN | -0.06 | 0.07 |
|  | Clinical Information | MDA5 |  | -0.06 | 0.07 |
|  | Glycan | Σ[aF_2] |  | -0.06 | 0.06 |
|  | IgG | Ig lambda-7 chain C region | LAC7_HUMAN | -0.06 | 0.14 |
|  | Protein | Hemopexin | HEMO_HUMAN | -0.06 | 0.15 |
|  | Clinical Information | CK |  | -0.07 | 0.07 |
|  | Protein | Alpha-1-antitrypsin | A1AT_HUMAN | -0.07 | 0.09 |
|  | IgG | Ig heavy chain V-III region GAR | HV322_HUMAN | -0.07 | 0.11 |
|  | Glycan | Σ[B_1] | Sum B | -0.08 | 0.14 |
|  | IgG | Ig heavy chain V-III region BUR | HV312_HUMAN | -0.08 | 0.14 |
|  | Protein | Apolipoprotein L1 | APOL1_HUMAN | -0.09 | 0.11 |
|  | Protein | Coagulation factor V | FA5_HUMAN | -0.11 | 0.13 |
|  | Protein | Apolipoprotein B-100 | APOB_HUMAN | -0.11 | 0.12 |
|  | Protein | Angiogenin | ANGI_HUMAN | -0.11 | 0.12 |
|  | Protein | Coagulation factor XI | FA11_HUMAN | -0.14 | 0.15 |

**Supplementary Table 7** Run-to-run variation for the control sample run 10 times in-between samples across the LC-MS/MS analysis. Glycan abbreviations are described in Figure 1

|  | IgG_1_ |  | IgG_2_ |  | IgG_34_ |  |
| --- | --- | --- | --- | --- | --- | --- |
|  | Average | STDV | Average | STDV | Average | STDV |
| FGlcNAc | 0.4% | 0.05% | 0.2% | 0.03% | 0.1% | 0.05% |
| A2 | 0.6% | 0.1% | 1% | 0.1% | - | - |
| A2B | 0.7% | 0.2% | 1% | 0.1% | 0.4% | 0.1% |
| A2G1 | 1.2% | 0.1% | - | - | - | - |
| A2G2 | 0.6% | 0.1% | - | - | - | - |
| FA1 | 0.6% | 0.1% | 1% | 0.1% | 0.4% | 0.1% |
| FA1G1 | 0.3% | 0.02% | 0.3% | 0.04% | 0.1% | 0.04% |
| FA1G1S1 | 0.2% | 0.07% | 0.1% | 0.02% | - | - |
| FA2 | 25% | 1% | 36% | 1% | 31% | 2% |
| FA2G1 | 29% | 1% | 31% | 1% | 26% | 1% |
| FA2G2 | 11% | 1% | 10% | 1% | 9% | 0.5% |
| FA2G1S1 | 2% | 1% | 2% | 0.2% | 3% | 1% |
| FA2G2S1 | 8% | 2% | 2% | 0.3% | 10% | 2% |
| FA2B | 8% | 0.5% | 9% | 1% | 11% | 1% |
| FA2BG1 | 10% | 1% | 6% | 0.2% | 7% | 0.3% |
| FA2BG2 | 1% | 0.2% | 0.4% | 0.05% | 1% | 0.2% |
| FA2BG1S1 | 0.3% | 0.1% | 0.02% | 0.02% | - | - |
| FA2BG2S1 | 0.2% | 0.1% | 0.04% | 0.02% | - | - |

**Supplementary Table 8** Glycopeptides sequences and glycan types that were screened for. Glycan abbreviations are described in Figure 1

| Peptide; IgG type; charges | Glycoforms |
| --- | --- |
| EEQYNSTYR: IgG1; 2+ and 3+ | GlcNAc |
|  | FGlcNAc |
| EEQFNSTFR: IgG2/3; 2+ and 3+ | A2 |
| EEQFNSTYR/EEQYNSTFR: IgG4/(3); 2+ and 3+ | A2B |
|  | A2G1 |
| TKPREEQYNSTYR: IgG1; 3+ and 4+ | A2G2 |
| TKPREEQFNSTFR: IgG2/3; 3+ and 4+ | FA1 |
| TKPREEQFNSTYR/EEQYNSTFR: IgG4/(3); 3+ and 4+ | FA1G1 |
|  | FA1G1S1 |
|  | FA2 |
|  | FA2G1 |
|  | FA2G2 |
|  | FA2G1S1 |
|  | FA2G2S1 |
|  | FA2G2S2 |
|  | FA2B |
|  | FA2BG1 |
|  | FA2BG2 |
|  | FA2BG1S1 |
|  | FA2BG2S1 |
|  | FA2BG2S2 |

**Supplementary Table 9** Factors included in the OPLS-DA model used to extract factors that could distinguish ASS/IIM and controls with 95% confidence. Glycan abbreviations are described in Figure 1 and in Table 3.

| **Correlation** | **Type** | **Variable** | **Uniprot ID** | **pq[1]** | **±95% confidence interval** |
| --- | --- | --- | --- | --- | --- |
| IIM correlating | Glycan | log(FA2_1/FA2_2) |  | 0.19 | 0.07 |
|  | IgG | Ig lambda chain V-III region SH | LV301_HUMAN | 0.18 | 0.11 |
|  | Glycan | Σ[aG_1] |  | 0.18 | 0.12 |
|  | IgG | Ig heavy chain V-II region ARH-77 | HV209_HUMAN | 0.17 | 0.14 |
|  | Glycan | Σ[aG_total] |  | 0.14 | 0.14 |
|  | Protein | Low affinity immunoglobulin gamma Fc region receptor III-A | FCG3A_HUMAN | 0.14 | 0.09 |
|  | IgG | Ig kappa chain V-III region GOL | KV307_HUMAN | 0.13 | 0.12 |
|  | IgG | Ig gamma-1 chain C region | IGHG1_HUMAN | 0.11 | 0.11 |
| Control correlating | Protein | Clusterin | CLUS_HUMAN | -0.21 | 0.10 |
|  | Protein | Platelet factor 4 | PLF4_HUMAN | -0.21 | 0.14 |
|  | Protein | Complement C1q subcomponent subunit B | C1QB_HUMAN | -0.19 | 0.11 |
|  | Glycan | Σ[G_1] |  | -0.17 | 0.11 |
|  | Protein | Titin | TITIN_HUMAN | -0.17 | 0.10 |
|  | Protein | Complement C1q subcomponent subunit C | C1QC_HUMAN | -0.16 | 0.09 |
|  | Protein | Serum albumin | ALBU_HUMAN | -0.16 | 0.14 |
|  | Protein | Complement C1q subcomponent subunit A | C1QA_HUMAN | -0.16 | 0.08 |
|  | Protein | Properdin | PROP_HUMAN | -0.15 | 0.07 |
|  | Protein | Apolipoprotein L1 | APOL1_HUMAN | -0.15 | 0.10 |
|  | Glycan | Σ[G_total] |  | -0.14 | 0.13 |
|  | Protein | Transthyretin | TTHY_HUMAN | -0.13 | 0.11 |
|  | IgG | Ig kappa chain V-I region Mev | KV120_HUMAN | -0.12 | 0.12 |
|  | IgG | Ig kappa chain V-III region NG9 | KV303_HUMAN | -0.10 | 0.09 |
| Other factors | IgG | Ig kappa chain V-I region EU | KV106_HUMAN | 0.15 | 0.18 |
|  | Glycan | Σ[aG_34] |  | 0.10 | 0.16 |
|  | IgG | Ig kappa chain V-I region WEA | KV118_HUMAN | 0.10 | 0.19 |
|  | Protein | Fibrinogen beta chain | FIBB_HUMAN | 0.10 | 0.13 |
|  | IgG | Ig lambda chain V-III region LOI | LV302_HUMAN | 0.10 | 0.15 |
|  | IgG | Ig kappa chain V-I region HK102 | KV110_HUMAN | 0.10 | 0.13 |
|  | IgG | Ig lambda chain V-VI region EB4 | LV605_HUMAN | 0.09 | 0.19 |
|  | Glycan | Σ[aG_2] |  | 0.09 | 0.16 |
|  | IgG | Immunoglobulin lambda-like polypeptide 5 | IGLL5_HUMAN | 0.09 | 0.18 |
|  | IgG | Ig kappa chain V-I region AG | KV101_HUMAN | 0.08 | 0.16 |
|  | IgG | Ig lambda chain V-V region DEL | LV501_HUMAN | 0.08 | 0.15 |
|  | Protein | CD5 antigen-like | CD5L_HUMAN | 0.08 | 0.20 |
|  | IgG | Ig lambda-2 chain C regions | LAC2_HUMAN | 0.08 | 0.10 |
|  | IgM | Ig mu chain C region | IGHM_HUMAN | 0.08 | 0.15 |
|  | IgM | Immunoglobulin J chain | IGJ_HUMAN | 0.07 | 0.19 |
|  | IgG | Ig heavy chain V-III region WEA | HV302_HUMAN | 0.07 | 0.17 |
|  | IgG | Ig lambda chain V-IV region Hil | LV403_HUMAN | 0.07 | 0.14 |
|  | Glycan | Σ[aF_2] |  | 0.07 | 0.18 |
|  | Protein | Complement factor H-related protein 1 | FHR1_HUMAN | 0.07 | 0.12 |
|  | IgG | Ig kappa chain V-I region AU | KV102_HUMAN | 0.07 | 0.20 |
|  | IgG | Ig kappa chain V-I region DEE | KV105_HUMAN | 0.07 | 0.18 |
|  | Protein | Haptoglobin | HPT_HUMAN | 0.06 | 0.20 |
|  | IgG | Ig heavy chain V-II region SESS | HV208_HUMAN | 0.06 | 0.20 |
|  | Glycan | Σ[aF_total] |  | 0.06 | 0.16 |
|  | IgG | Ig heavy chain V-I region HG3 | HV102_HUMAN | 0.06 | 0.12 |
|  | Protein | Complement factor B | CFAB_HUMAN | 0.05 | 0.14 |
|  | IgG | Ig lambda chain V-II region TRO | LV204_HUMAN | 0.05 | 0.20 |
|  | IgG | Ig kappa chain V-I region Scw | KV117_HUMAN | 0.05 | 0.13 |
|  | IgG | Ig heavy chain V-III region BUR | HV312_HUMAN | 0.05 | 0.09 |
|  | IgG | Ig heavy chain V-III region KOL | HV311_HUMAN | 0.05 | 0.14 |
|  | Protein | Apolipoprotein E | APOE_HUMAN | 0.05 | 0.10 |
|  | IgG | Immunoglobulin lambda-like polypeptide 1 | IGLL1_HUMAN | 0.04 | 0.17 |
|  | Protein | Complement component C8 gamma chain | CO8G_HUMAN | 0.04 | 0.09 |
|  | Glycan | Σ[aF_1] |  | 0.04 | 0.15 |
|  | Protein | Complement component C8 beta chain | CO8B_HUMAN | 0.04 | 0.18 |
|  | IgG | Ig heavy chain V-II region WAH | HV206_HUMAN | 0.04 | 0.21 |
|  | IgG | Ig kappa chain V-III region SIE | KV302_HUMAN | 0.03 | 0.15 |
|  | Protein | Fibrinogen alpha chain | FIBA_HUMAN | 0.03 | 0.19 |
|  | IgG | Ig lambda chain V-I region BL2 | LV107_HUMAN | 0.03 | 0.16 |
|  | IgG | Ig kappa chain V-I region HK101 | KV109_HUMAN | 0.02 | 0.16 |
|  | IgD | Ig delta chain C region | IGHD_HUMAN | 0.02 | 0.17 |
|  | IgG | Ig lambda chain V-I region VOR | LV101_HUMAN | 0.02 | 0.18 |
|  | IgG | Ig lambda chain V-I region NEW | LV103_HUMAN | 0.02 | 0.10 |
|  | Protein | Plasma protease C1 inhibitor | IC1_HUMAN | 0.02 | 0.17 |
|  | IgG | Ig kappa chain V-I region Walker | KV123_HUMAN | 0.01 | 0.20 |
|  | IgG | Ig kappa chain V-I region Kue | KV112_HUMAN | 0.01 | 0.19 |
|  | IgG | Ig kappa chain V-I region Roy | KV116_HUMAN | 0.01 | 0.16 |
|  | Protein | Coagulation factor V | FA5_HUMAN | 0.00 | 0.20 |
|  | Protein | Apolipoprotein C-I | APOC1_HUMAN | 0.00 | 0.13 |
|  | Clinical Information | Age |  | 0.00 | 0.06 |
|  | Protein | Gelsolin | GELS_HUMAN | 0.00 | 0.11 |
|  | Clinical Information | Male |  | 0.00 | 0.18 |
|  | Ig3 | Ig gamma-3 chain C region | IGHG3_HUMAN | 0.00 | 0.10 |
|  | Protein | Fibronectin | FINC_HUMAN | 0.00 | 0.11 |
|  | IgG | Ig heavy chain V-III region CAM | HV307_HUMAN | 0.00 | 0.10 |
|  | Clinical Information | Female |  | 0.00 | 0.18 |
|  | Glycan | Σ[B_1] |  | 0.00 | 0.16 |
|  | IgG | Ig kappa chain V-I region | KV104_HUMAN | 0.00 | 0.14 |
|  | IgG | Ig heavy chain V-III region LAY | HV314_HUMAN | 0.00 | 0.12 |
|  | IgG | Ig kappa chain V-III region B6 | KV301_HUMAN | 0.00 | 0.14 |
|  | Protein | Fibrinogen gamma chain | FIBG_HUMAN | 0.00 | 0.18 |
|  | IgG | Ig kappa chain V-II region MIL | KV203_HUMAN | -0.01 | 0.16 |
|  | IgG | Ig heavy chain V-III region GA | HV308_HUMAN | -0.01 | 0.15 |
|  | IgG | Ig lambda chain V region 4A | LV001_HUMAN | -0.01 | 0.18 |
|  | Protein | Apolipoprotein B-100 | APOB_HUMAN | -0.01 | 0.17 |
|  | Protein | Dermcidin | DCD_HUMAN | -0.01 | 0.13 |
|  | IgM | Ig mu heavy chain disease protein | MUCB_HUMAN | -0.01 | 0.12 |
|  | IgG | Ig kappa chain V-III region IARC/BL41 | KV311_HUMAN | -0.02 | 0.14 |
|  | IgA | Ig alpha-1 chain C region | IGHA1_HUMAN | -0.02 | 0.17 |
|  | Protein | Angiogenin | ANGI_HUMAN | -0.02 | 0.19 |
|  | IgG | Ig heavy chain V-III region JON | HV319_HUMAN | -0.02 | 0.14 |
|  | Glycan | Σ[B_total] |  | -0.02 | 0.11 |
|  | IgG | Ig heavy chain V-III region GAR | HV322_HUMAN | -0.02 | 0.13 |
|  | Glycan | Σ[B_34] |  | -0.02 | 0.11 |
|  | Protein | Vitronectin | VTNC_HUMAN | -0.03 | 0.16 |
|  | Glycan | Σ[B_2] |  | -0.03 | 0.10 |
|  | IgG | Ig heavy chain V-III region BUT | HV306_HUMAN | -0.03 | 0.15 |
|  | Protein | Kininogen-1 | KNG1_HUMAN | -0.03 | 0.11 |
|  | Protein | Complement C4-B | CO4B_HUMAN | -0.03 | 0.11 |
|  | IgG | Ig kappa chain V-I region Wes | KV119_HUMAN | -0.03 | 0.09 |
|  | Glycan | Σ[S_2] |  | -0.03 | 0.10 |
|  | IgG | Ig kappa chain V-III region VG | KV309_HUMAN | -0.03 | 0.18 |
|  | Protein | Ribonuclease 4 | RNAS4_HUMAN | -0.04 | 0.12 |
|  | Protein | Complement factor H-related protein 5 | FHR5_HUMAN | -0.04 | 0.09 |
|  | Protein | Prothrombin | THRB_HUMAN | -0.04 | 0.10 |
|  | Protein | Hemopexin | HEMO_HUMAN | -0.04 | 0.19 |
|  | Protein | Antithrombin-III | ANT3_HUMAN | -0.04 | 0.13 |
|  | IgG | Ig heavy chain V-III region WAS | HV315_HUMAN | -0.04 | 0.12 |
|  | Protein | Vitamin K-dependent protein S | PROS_HUMAN | -0.04 | 0.12 |
|  | IgG | Ig heavy chain V-I region V35 | HV103_HUMAN | -0.04 | 0.13 |
|  | Protein | Beta-2-glycoprotein 1 | APOH_HUMAN | -0.04 | 0.14 |
|  | IgG | Ig heavy chain V-III region GAL | HV320_HUMAN | -0.04 | 0.11 |
|  | Glycan | Σ[aF_34] |  | -0.04 | 0.11 |
|  | Protein | Complement factor I | CFAI_HUMAN | -0.04 | 0.10 |
|  | Protein | C4b-binding protein beta chain | C4BPB_HUMAN | -0.04 | 0.17 |
|  | Protein | Thrombospondin-1 | TSP1_HUMAN | -0.04 | 0.11 |
|  | IgG | Ig kappa chain V-II region FR | KV202_HUMAN | -0.04 | 0.17 |
|  | IgG | Ig kappa chain V-IV region Len | KV402_HUMAN | -0.05 | 0.12 |
|  | IgG | Ig heavy chain V-III region TRO | HV301_HUMAN | -0.05 | 0.17 |
|  | Protein | Coagulation factor XI | FA11_HUMAN | -0.05 | 0.23 |
|  | Protein | Inter-alpha-trypsin inhibitor heavy chain H4 | ITIH4_HUMAN | -0.05 | 0.16 |
|  | Protein | Complement factor H | CFAH_HUMAN | -0.05 | 0.17 |
|  | IgG | Ig kappa chain V-I region BAN | KV122_HUMAN | -0.05 | 0.14 |
|  | KC | Ig kappa chain C region | IGKC_HUMAN | -0.06 | 0.19 |
|  | IgG | Ig lambda chain V-I region WAH | LV106_HUMAN | -0.06 | 0.16 |
|  | Protein | Protein AMBP | AMBP_HUMAN | -0.06 | 0.09 |
|  | IgG | Ig kappa chain V-I region Lay | KV113_HUMAN | -0.06 | 0.16 |
|  | LC | Ig lambda-7 chain C region | LAC7_HUMAN | -0.06 | 0.11 |
|  | Protein | C4b-binding protein alpha chain | C4BPA_HUMAN | -0.06 | 0.13 |
|  | Protein | Alpha-2-macroglobulin | A2MG_HUMAN | -0.07 | 0.23 |
|  | IgA | Ig alpha-2 chain C region | IGHA2_HUMAN | -0.07 | 0.18 |
|  | IgG | Ig heavy chain V-III region VH26 | HV303_HUMAN | -0.07 | 0.12 |
|  | Protein | Serotransferrin | TRFE_HUMAN | -0.07 | 0.15 |
|  | Protein | Complement C5 | CO5_HUMAN | -0.07 | 0.09 |
|  | Protein | Plasminogen | PLMN_HUMAN | -0.08 | 0.21 |
|  | IgG | Ig gamma-4 chain C region | IGHG4_HUMAN | -0.09 | 0.16 |
|  | Protein | Platelet basic protein | CXCL7_HUMAN | -0.09 | 0.12 |
|  | Protein | Histidine-rich glycoprotein | HRG_HUMAN | -0.09 | 0.13 |
|  | Protein | Complement C2 | CO2_HUMAN | -0.09 | 0.10 |
|  | Glycan | Σ[G_34] |  | -0.09 | 0.15 |
|  | IgG | Ig gamma-2 chain C region | IGHG2_HUMAN | -0.09 | 0.14 |
|  | Protein | Complement C4-A | CO4A_HUMAN | -0.10 | 0.10 |
|  | Protein | Putative V-set and immunoglobulin domain-containing-like protein IGHV4OR15-8 | IV4F8_HUMAN | -0.10 | 0.13 |
|  | Protein | Complement C1r subcomponent-like protein | C1RL_HUMAN | -0.10 | 0.15 |
|  | Protein | Alpha-1-antitrypsin | A1AT_HUMAN | -0.10 | 0.17 |
|  | IgG | Ig heavy chain V-III region BRO | HV305_HUMAN | -0.10 | 0.15 |
|  | Protein | Complement C3 | CO3_HUMAN | -0.10 | 0.14 |
|  | Glycan | Σ[G_2] |  | -0.10 | 0.16 |
|  | Protein | Complement C1s subcomponent | C1S_HUMAN | -0.11 | 0.18 |
|  | Glycan | Σ[S_34] |  | -0.11 | 0.12 |
|  | Protein | Apolipoprotein A-I | APOA1_HUMAN | -0.11 | 0.12 |
|  | Glycan | Σ[S_total] |  | -0.12 | 0.12 |
|  | Protein | Complement C1r subcomponent | C1R_HUMAN | -0.12 | 0.15 |
|  | Protein | Haptoglobin-related protein | HPTR_HUMAN | -0.12 | 0.15 |
|  | Protein | Neutrophil defensin 3 | DEF3_HUMAN | -0.12 | 0.12 |
|  | Protein | Lysozyme C | LYSC_HUMAN | -0.12 | 0.13 |
|  | Glycan | Σ[S_1] |  | -0.12 | 0.17 |

**Supplementary Figure 1**


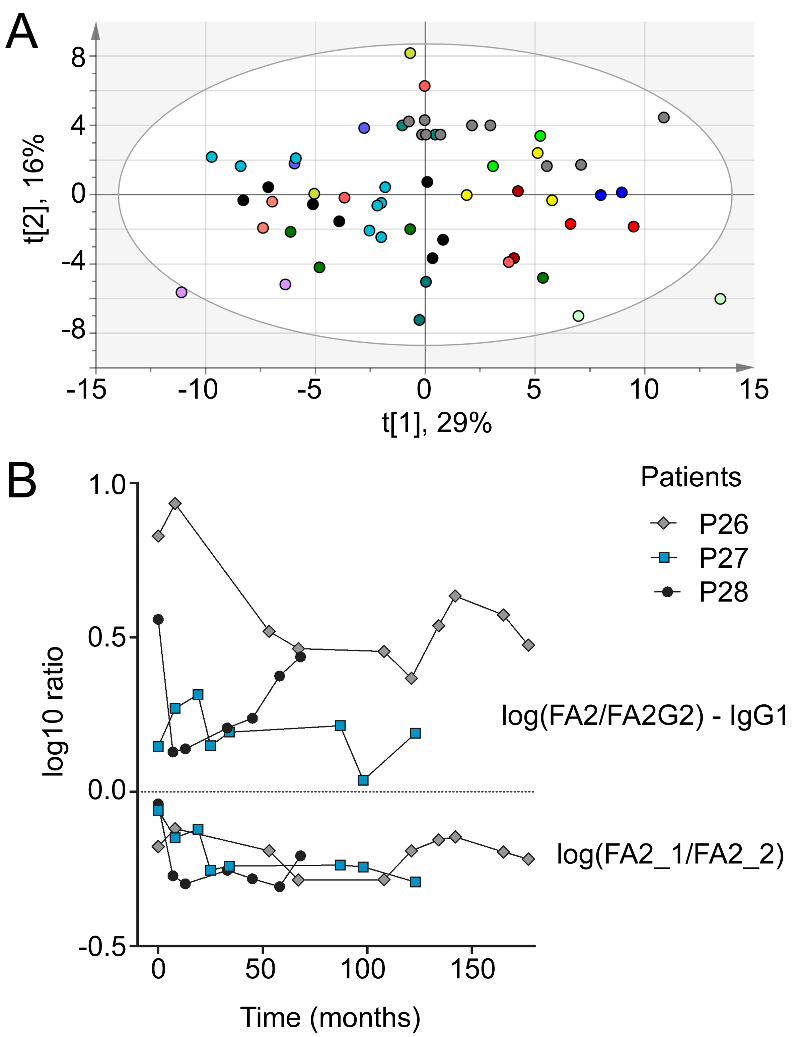


**Supplementary Figure 1.** Intra-individual variation in glycan profiles according to time. **(A)** PCA analysis of the Fc-glycan profiles of the patients sampled at more than one time point (T1-T10), described in Supplemental Table 1. As shown in the scores plot, the Fc-glycan profiles from the same patients (dots with the same color represents one individual) clusters together in the same part of the plot, indicating that the profiles are similar over time. (**B**) The log(FA2_1/FA2_2) ratio and the log(FA2/FA2G2) ratio of IgG_1_ measured over time from the three patients with most available sampling data (T1-T7, T1-T8 and T1-T10), respectively. No distinct trend is observed over time for the two markers. However, log(FA2_1/FA2_2) is more stable over time, likely since it is less effected by other factors that will also affect the galactosylation status of the N-glycome. Thus, log(FA2_1/FA2_2) is likely a better disease specific marker than log(FA2/FA2G2).

**Supplementary Figure 2**

**Supplementary Figure 2.** Diagram of the different IgG subtypes purified from IIM (Jo1^+^ and Jo1^-^) and HC sera **(A)** (Adapted from Ossipova *et al* 2014).^2^ Anti-Jo1-IgG ELISA to confirm reactivity of anti-Jo1 IgG eluted from the Jo1-affinity column (average of duplicates from 3 different experiments). Dash line represents OD displayed by non-Jo1 reactive IgG (Jo1FT) **(B)**. Sodium dodecyl sulfate polyacrylamide gel electrophoresis (SDS-PAGE) image of protein fractions collected throughout anti-Jo1 IgG purification (**C upper and lower panels**). In the upper panel (**C upper**) affinity purification fractions from HC, Jo1^-^ IIM and Jo1^+^ IIM individuals were run onto a SDS-PAGE gel. S, serum from HC, Jo1^-^ IIM and Jo1^+^ IIM; PGFT, protein G flow through = serum-depleted IgG. IgG, total IgG eluted from the protein G column; Jo1FT, non-Jo1 reactive IIM IgG collected from the Jo1 affinity column. Jo1IgG, anti-Jo1 reactive IgG eluted from the Jo1 affinity column. Because additional bands were detected in the molecular weight corresponding to the light and heavy chain of anti-Jo1 IgG one extra gel was loaded with fractions from anti-Jo1 IgG purification of another Jo1^+^ IIM patient (**C lower panel)**. **(D)** Dot-blot image illustrating reactivity to the recombinant (r)Jo1 limited to IIM Jo1^+^ serum (second square), total Jo1^+^ IIM IgG (forth square), and anti-Jo1 IgG fraction (sixth square). rJo1, nitrocellulose membrane containing recombinant (r)Jo1 incubated with commercial anti-Jo1 antibody. 2°Ab, nitrocellulose membrane-containing rJo1 uniquely incubated with the secondary anti-mouse antibody. Full-length gels are displayed. Acquisition of gels and dot-blot images was performed in a standard scanner.

**Supplementary Figure 3**





**Supplementary Figure 3.** Log scale abundance of the two factors (bisected, B and afucosylated, aF forms) that were best at distinguishing the Jo1^+^ and Jo1^-^ patients. **(A)** The sum of bisected forms characterized in all isotypes, i.e. Σ[B_total]. **(B)** The sum of bisected forms of IgG_2_, i.e. Σ[B_2]. **(C)** The sum of afucosylated forms characterized in all isotypes, i.e. Σ[aF_total]. (**D**) The sum of afucosylated forms of IgG_1_, i.e. Σ[aF_1]. In addition to the controls and the Jo1^+^ and Jo1^-^ patients, the values obtained from anti-Jo1 specific IgG and paired FTs are shown. Values obtained for IIM ILD and/or ASS diagnosed patients are colored in black. Indicated numbers are p-values, ns: not significant. P-values <0.005 remain significant following FDR correction.

**Supplementary Figure 4**


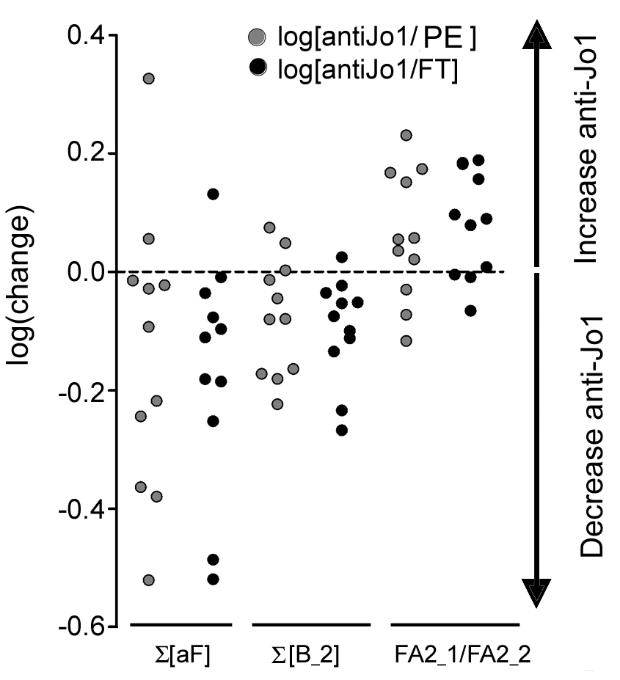


**Supplementary Figure 4.** Log10 fold change of the intra-individual anti-Jo1/FT and anti-Jo1/PE ratios. Values below zero indicate a decrease in anti-Jo1 specific IgG relative to the matched FT or pre-enrichment total anti-Jo1^+^ IgG pools (PE). Values above zero indicate an increase.

**Supplementary Figure 5**


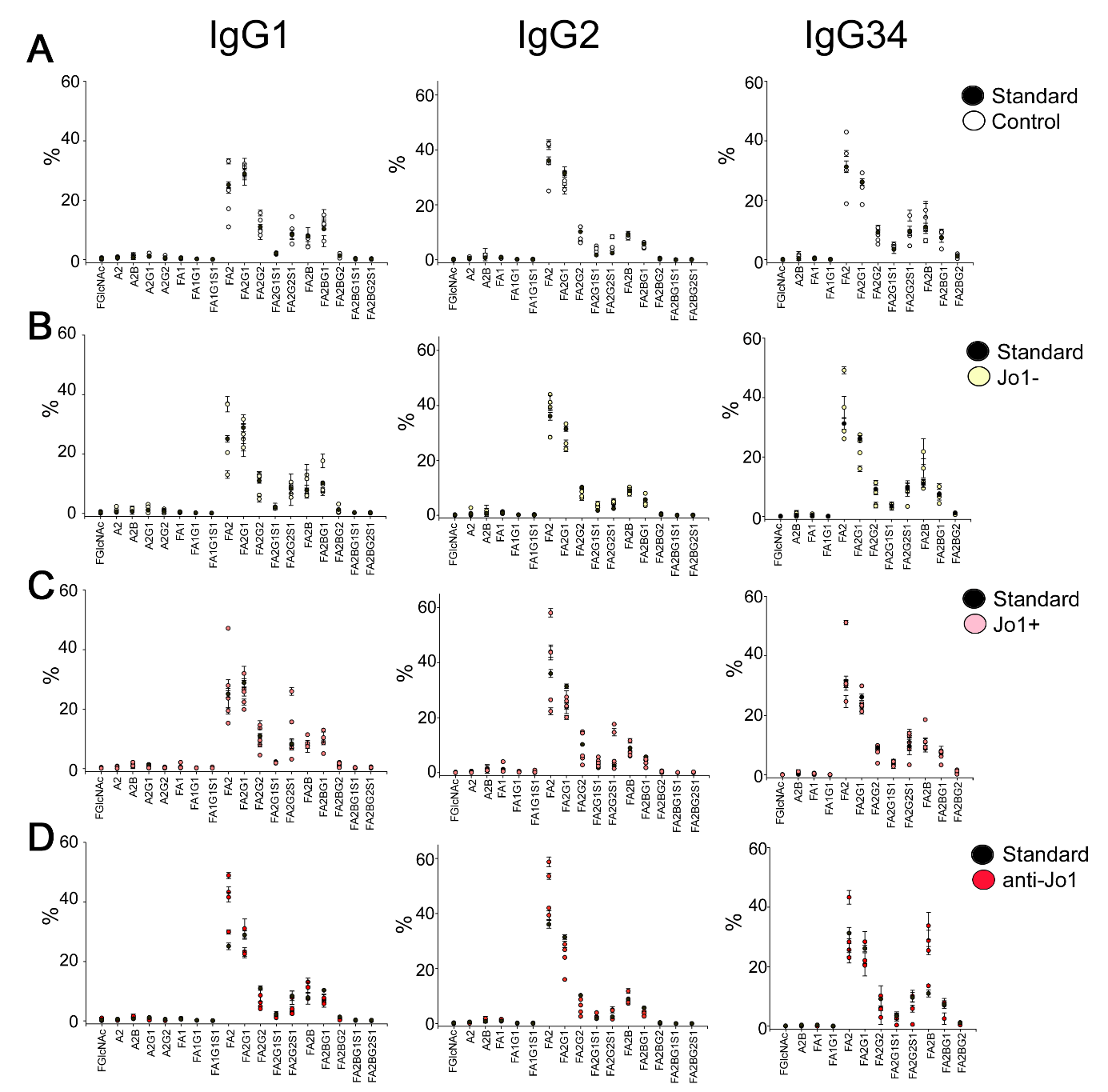
**Supplementary Figure 5.** Variation between the IgG digests (average and standard deviation) of **A**) four healthy control IgG, **B**) four Jo1^-^ patient IgG, **C**) five Jo1^+^ patient IgG and **D**) four anti-Jo1 IgG specific samples.

**Supplementary Figure 6.**


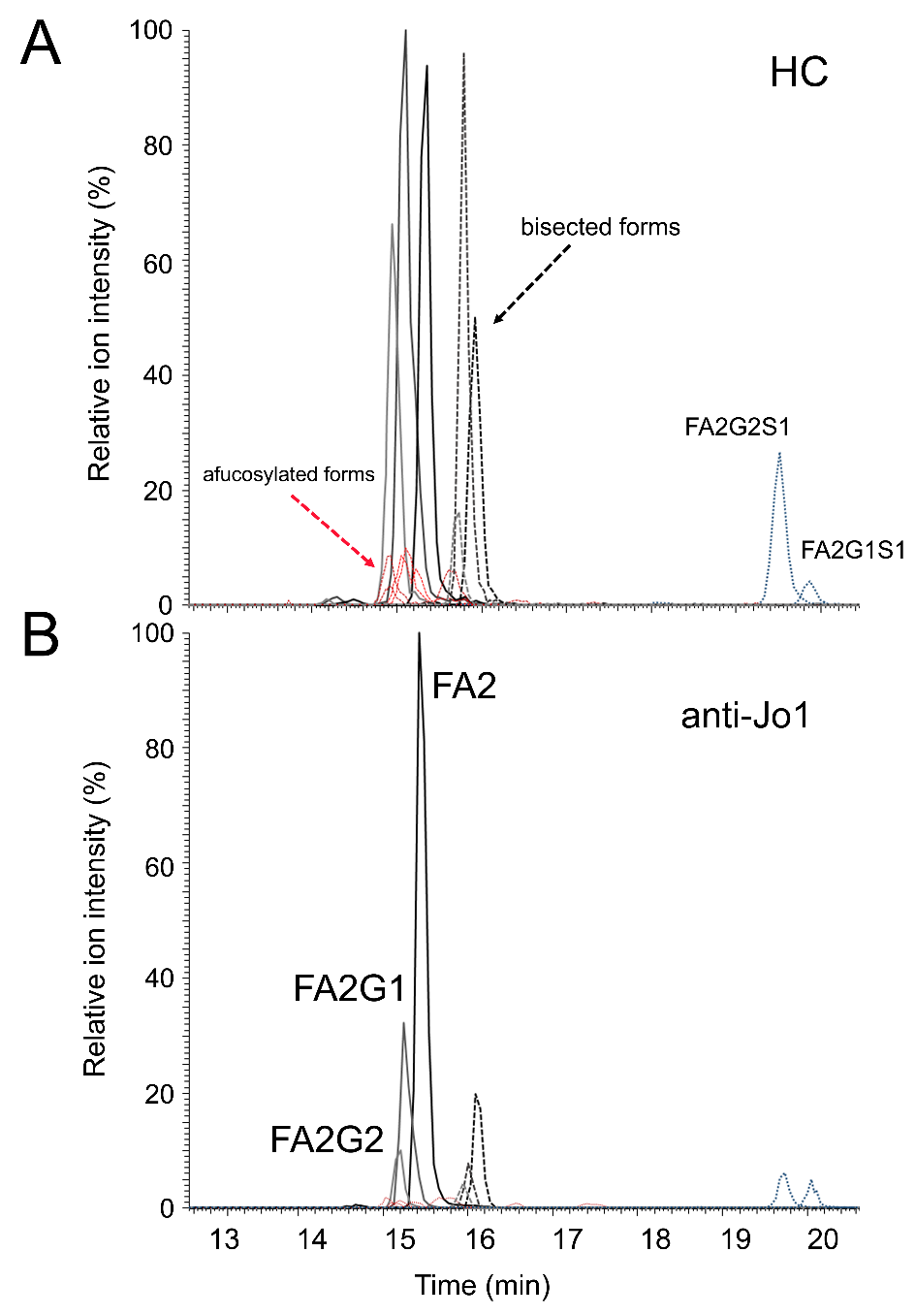


**Supplementary Figure 6.** Extracted ion chromatograms of the more abundant glycoforms from combined doubly and triply charged ions of glycopeptide EEQYNSTYR (from IgG_1_). A) Healthy control IgG. B) Anti-Jo1 specific IgG. Note the big differences in FA2 (more abundant) and in the bisected and afucosylated forms (less abundant), compared to the healthy control sample.

**Supplementary Figure 7**


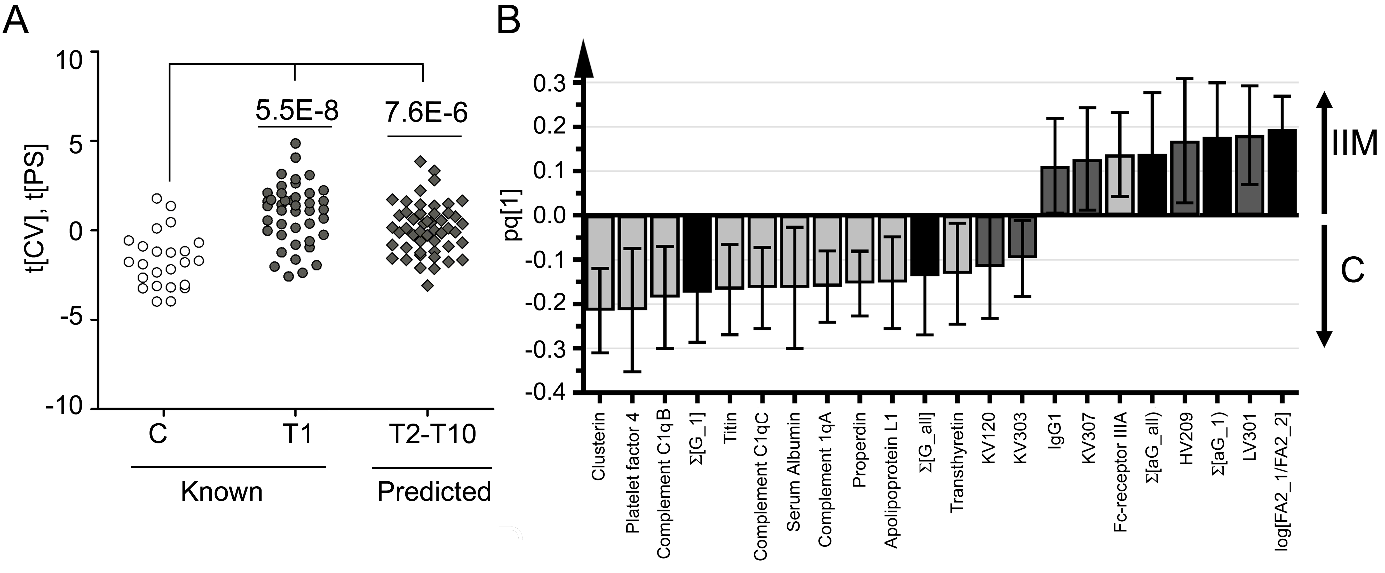


**Supplementary Figure 7.** Multivariate correlation analyses according to ASS/IIM. **A)** Dot plot of the cross validated (tCV) scores (circles) of and predicted scores (diamonds) obtained from a model set to identify ASS/IIM correlating features in the data set (containing the Fc-glycan profiles, samples protein content and clinical factors, listed in Supplementary Table 9). Both the IIM profiles of the patients that were treated as known (i.e. from which the model was based of) and unknown (the validation cohort of the patients at timepointsT2-T10), are significantly different compared to the controls. **B)** The factors that correlated positively or negatively with ASS/IIM with 95% confidence.

**References**

1 Lyutvinskiy, Y., Yang, H., Rutishauser, D. & Zubarev, R. A. In silico instrumental response correction improves precision of label-free proteomics and accuracy of proteomics-based predictive models. *Mol Cell Proteomics* **12**, 2324-2331, doi:10.1074/mcp.O112.023804 (2013).

2 Ossipova, E. *et al.* Affinity purified anti-citrullinated protein/peptide antibodies target antigens expressed in the rheumatoid joint. *Arthritis Res Ther* **16**, R167, doi:10.1186/ar4683 (2014).
